# Anti-tat Hutat2:Fc mediated protection against tat-induced neurotoxicity and HIV-1 replication in human monocyte-derived macrophages

**DOI:** 10.1186/s12974-014-0195-2

**Published:** 2014-11-22

**Authors:** Wen Kang, Wayne A Marasco, Hsin-I Tong, Mary Margaret Byron, Chengxiang Wu, Yingli Shi, Si Sun, Yongtao Sun, Yuanan Lu

**Affiliations:** Department of Infectious Diseases, Tangdu Hospital, The Fourth Military Medical University, 569 Xinsi Road, Xi’an, Shaanxi 710038 China; Department of Public Health Sciences, John A. Burns School of Medicine, University of Hawaii, 1960 East–west Road, Honolulu, HI 96822 USA; Department of Cancer Immunology and AIDS, Dana-Farber Cancer Institute, Harvard Medical School, 50 Brookline Avenue, Boston, MA 02215 USA; Hawaii Center for AIDS, John A. Burns School of Medicine, University of Hawaii, 651 Ilalo St., BSB, Suite 231, Honolulu, HI 96813 USA

**Keywords:** Anti-Tat antibody, HIV-1, HIV-associated neurocognitive disorders, Human monocyte-derived macrophages, Lentivirus, Neuroprotection

## Abstract

**Background:**

HIV-1 Tat is essential for HIV replication and is also a well-known neurotoxic factor causing HIV-associated neurocognitive disorder (HAND). Currently, combined antiretroviral therapy targeting HIV reverse transcriptase or protease cannot prevent the production of early viral proteins, especially Tat, once HIV infection has been established. HIV-infected macrophages and glial cells in the brain still release Tat into the extracellular space where it can exert direct and indirect neurotoxicity. Therefore, stable production of anti-Tat antibodies in the brain would neutralize HIV-1 Tat and thus provide an effective approach to protect neurons.

**Methods:**

We constructed a humanized anti-Tat Hutat2:Fc fusion protein with the goal of antagonizing HIV-1 Tat and delivered the gene into cell lines and primary human monocyte-derived macrophages (hMDM) by an HIV-based lentiviral vector. The function of the anti-Tat Hutat2:Fc fusion protein and the potential side effects of lentiviral vector-mediated gene transfer were evaluated *in vitro*.

**Results:**

Our study demonstrated that HIV-1-based lentiviral vector-mediated gene transduction resulted in a high-level, stable expression of anti-HIV-1 Tat Hutat2:Fc in human neuronal and monocytic cell lines, as well as in primary hMDM. Hutat2:Fc was detectable in both cells and supernatants and continued to accumulate to high levels within the supernatant. Hutat2:Fc protected mouse cortical neurons against HIV-1 Tat_86_-induced neurotoxicity. In addition, both secreted Hutat2:Fc and transduced hMDM led to reducing HIV-1_BaL_ viral replication in human macrophages. Moreover, lentiviral vector-based gene introduction did not result in any significant changes in cytomorphology and cell viability. Although the expression of *IL8*, *STAT1*, and *IDO1* genes was up-regulated in transduced hMDM, such alternation in gene expression did not affect the neuroprotective effect of Hutat2:Fc.

**Conclusions:**

Our study demonstrated that lentivirus-mediated gene transfer could efficiently deliver the *Hutat2:Fc* gene into primary hMDM and does not lead to any significant changes in hMDM immune-activation. The neuroprotective and HIV-1 suppressive effects produced by Hutat2:Fc were comparable to that of a full-length anti-Tat antibody. This study provides the foundation and insights for future research on the potential use of Hutat2:Fc as a novel gene therapy approach for HAND through utilizing monocytes/macrophages, which naturally cross the blood-brain barrier, for gene delivery.

**Electronic supplementary material:**

The online version of this article (doi:10.1186/s12974-014-0195-2) contains supplementary material, which is available to authorized users.

## Background

HIV-associated neurocognitive disorders (HAND) occur when HIV enters the central nervous system (CNS) and impairs neuronal function involved in cognitions, including memory, learning, attention, problem solving, and decision making [1]. It can be classified into three categories, namely asymptomatic neurocognitive impairment, mild neurocognitive disorder, and HIV-associated dementia [1,2]. Although more severe forms of HAND are rare thanks to the introduction of combined antiretroviral therapy (cART), the prevalence of milder forms continues to increase [3]. Cross-sectional studies showed that neurologic disorders occur in half of HIV-1-infected individuals in the era of cART [3-5]. One explanation for the high prevalence of HAND in the cART era is that cART cannot completely inhibit HIV replication in the CNS, which results in persistent HIV replication at low-levels in the brain [1,6-8]. Either prolonged toxic inflammatory activation or the presence of toxic viral products, such as the HIV-1 Tat protein produced by low-level HIV within the CNS, can continue to drive neurodegeneration [1,9]. Although several antiretroviral agents have been found to enter the CNS with higher efficiency, another issue that arises with cART for HAND treatment is the lack of a correlation between the CNS penetration-effectiveness (CPE) index and efficacy of therapy [10]. A recent study has shown that HIV dementia was even worse among affected individuals who received a cART regimen with a high CPE score [11]. Therefore, development of adjuvant therapies for HAND is urgently needed.

Gene therapy is currently being explored for combating HIV-1 infection [12]. There are numerous anti-HIV gene therapy approaches, all of which can be classified into three broad categories: genetic vaccine-based strategies utilizing HIV peptides or gene products [13-15]; RNA-based strategies including anti-sense RNA, RNA decoys (sense RNA), ribozymes, RNA aptamers, mutant tRNA_3_^Lys^, small interfering RNAs, and microRNAs [16-19]; and protein-based strategies including transdominant negative proteins, chimeric proteins (fusion proteins), nucleases, anti-infective cellular proteins, single-chain variable fragment intrabodies (scFv), and monoclonal antibodies [17,20-23]. Although a combined approach utilizing both mRNA and protein-based strategies would be more effective for HAND therapy, each strategy should be tested independently. It is presently known that vaccine-based strategies expressing HIV-1 proteins are not suitable for the treatment of HAND since these proteins are neurotoxic. Although RNA-based strategies interfere with intracellular gene expression, they offer no protection against existing extracellular neurotoxic HIV-1 proteins and inflammatory cytokines in the CNS. Therefore, protein-based gene therapy strategies targeting on both the intra- and extra-cellular neurotoxins would be helpful. Based on this hypothesis, we have developed a lentiviral vector-based gene transfer system to deliver the genes of secretory human brain-derived neurotrophic factor and soluble tumor necrosis factor-α receptor:Fc fusion protein into cell lines and primary monocyte-derived macrophages (MDM). These integrated genes could be expressed with high efficiency and have been shown to protect against TNF-α and HIV-1 Tat and gp120-induced neurotoxicity [24,25]. However, these two candidates are limited in their ability to inhibit HIV-1 replication directly.

HIV-1 Tat is a conserved non-structural protein that is essential for HIV-1 replication [26]. It can be secreted by HIV-1 infected macrophages and glial cells within the CNS, or easily enter the CNS by crossing the blood-brain barrier (BBB). Tat functions as a potent neurotoxin causing HAND directly and indirectly in the brain [27-30]. For example, Tat injures neurons directly through the dysregulation of intracellular Ca^2+^ levels, increasing excitotoxicity, and disinhibiting permeable N-methyl-D-aspartate receptors from Zn^2+^-mediated antagonism [31-33]. In addition, extracellular Tat can cause neuronal damage indirectly by increasing the expression of nitric oxide synthase and the release of toxins including nitric oxide (NO), TNF-α, and IL-1β from monocytes, macrophages, glial cells, and brain endothelial cells [28,34-36]. Therefore, any efforts to blunt the Tat effects would be expected to have profound and significant impact in treating HIV neuropathogenesis, decreasing the prevalence of HIV-associated neurological diseases and improving the quality of life of HIV-infected individuals. Previous attempts utilizing retrovirus-mediated gene transfer of a humanized anti-Tat intrabody termed as Hutat2 into CD4^+^ T cells have shown to successfully inhibit HIV-1 replication in infected mammalian cell lines and transduced CD4^+^ mononuclear cell populations [37-39]. Furthermore, a recent *in vivo* study indicated that retrovirus-mediated anti-Tat scFv Hutat2 transduction increased the relative survival of transduced CD4^+^ T cells infected with chimeric simian immunodeficiency virus/HIV, and was associated with a viral load reduction in one rhesus macaque [22].

This study is designed to explore the protective effects of lentiviral-mediated gene transfer of anti-Tat Hutat2:Fc against Tat-activated viral transcription as well as Tat-induced neurotoxicity. We modified the native anti-Tat Hutat2 sequence and constructed an HIV-1-based lentiviral vector HR-Hutat2, which expresses humanized anti-Tat scFv:Fc fusion protein (Hutat2:Fc) under the control of the human cytomegalovirus (CMV) promoter. This vector was shown to transduce human cell lines of both neuron and monocyte origins, as well as primary human MDMs (hMDM), resulting in the secretion of Hutat2:Fc fusion protein, albeit to varying levels. The secreted Hutat2:Fc was shown to be protective to mouse primary neurons that were exposed to HIV-1 Tat. In addition, both secreted Hutat2:Fc and HR-Hutat2-transduced hMDM led to prevention from Tat-activated HIV-1 transcription, thus suppressing viral replication and reducing the spread of viral infection in human macrophages. Potential adverse effects due to the lentiviral vector transduction were also evaluated by assessing the expression profiling of 15 macrophage-related functional and regulatory genes using a real-time PCR assay. Our findings lay out the groundwork for future studies using anti-Tat *Hutat2* gene-modified MDM as a potential therapeutic strategy for HAND.

## Methods

### Animal care

Balb/c mice were obtained from Dr. Federick Mercier, University of Hawaii at Manoa, USA. All mice were bred and maintained in the animal facility of the University of Hawaii at Manoa following institutional guidelines. All procedures were reviewed and approved by the University of Hawaii Animal Care and Use Committee and conducted according to the Animal Welfare Act and National Institutes of Health guidelines.

### Generation and production of the lentiviral vectors

A transfer plasmid containing an expression cassette for Hutat2:Fc fusion protein was constructed (Additional file 1). Briefly, the gene encoding the anti-HIV-1 Tat scFv Hutat2 with a leader sequence fused to the hinge domain from the human *IgG1* gene and the Fc domain from the human *IgG3* gene was commercially synthesized (GeneArt®, Life Technologies, Grand Island, NY, USA). The synthetic gene was amplified by PCR, using primer pairs containing Xho I and BamH I restriction sites (Additional file 1), and inserted into the backbone of pHR-HB7-IRES-GFP plasmid (generously provided by Dr. V. Planelles, University of Utah) that was digested with the same enzymes. The final bicistronic plasmid construct, pHR-Hutat2:Fc-EGFP, co-expressed the Hutat2:Fc fusion protein under a CMV promoter and the enhanced green fluorescent protein (EGFP) via the internal ribosome entry site (IRES) element. Another transfer plasmid containing an expression cassette for anti-Epstein-Barr virus latent membrane protein 1 scFv (A3H5:Fc) was constructed in the same way and used as a control. Lentiviral vectors encoding the Hutat2:Fc (*HR-Hutat2*) or control (*HR-A3H5*) genes were generated by transient co-transfection in 293 T cells with pCMV-ΔR8.2 and pCMV-VSV-G. Vector production and concentration were performed as described previously [40-42]; 293 T cells were used for vector titration [25]. High-titer lentiviral vector stocks (3.3 to 4.8 × 10^8^ U/mL) were prepared by sucrose-cushioned ultracentrifugation at 25,000 rpm for 2 hours [40].

### Cell lines and culture

Human embryonic kidney 293 T cells (GenHunter Co., Nashville, TN, USA) were maintained in Dulbecco’s Modified Eagle’s Medium (Corning Life Sciences, Manassas, VA, USA) supplemented with 1.0 g/L glucose, 4 mM L-glutamine (Sigma-Aldrich, St. Louis, MO, USA), 1.0 mM sodium pyruvate (Corning Life Sciences), 100 IU/mL penicillin (Sigma-Aldrich), 0.1 mg/mL streptomycin (Sigma-Aldrich), 10 mM HEPES (HyClone, South Logan, UT, USA), and 10% fetal bovine serum (FBS) (HyClone). The human neuroblastoma cell line HTB-11 (ATCC, Manassas, VA, USA), was cultured in Minimum Essential Medium (Eagle) (Corning Life Sciences) supplemented with 2 mM L-glutamine, 1.0 mM sodium pyruvate, 100 IU/mL penicillin, 0.1 mg/mL streptomycin, and 10% FBS. Culture media was replaced every 2 to 3 days and cells were sub-cultured with EDTA solution containing 0.25% trypsin (Sigma-Aldrich). The human monocytic cell line U937 (ATCC) was cultured in RPMI 1640 (Sigma-Aldrich) supplemented with 2 mM L-glutamine, 1.0 mM sodium pyruvate, 100 IU/mL penicillin, 0.1 mg/mL streptomycin, and 10% FBS. Cells were maintained at 37°C in 5% CO_2_.

### Isolation and cultivation of hMDM

Human peripheral blood was anonymously collected from three blood donors (serum negative for HIV-1, hepatitis B, and hepatitis C) with specific approval of the University of Hawaii’s Institutional Review Board (UH IRB). Signed consent forms were received from blood donors and the procedures for blood collection and usage were reviewed and approved by the UH IRB. Peripheral blood mononuclear cells were isolated using Ficoll-Paque™ Plus (GE Healthcare Biosciences, Piscataway, NJ, USA) and plated at a density of 3.5 × 10^6^ per well in RPMI 1640 medium supplemented with 10% defined FBS (HyClone), 1% sodium pyruvate, 100 IU/mL penicillin, 0.1 mg/mL streptomycin, and 1,000 U/mL human macrophage colony stimulating factor (M-CSF; obtained from 5/9 m α3-18 cell conditioned medium, ATCC#CRL-10154) using 12-well plates, and incubated at 37°C in 5% CO_2_. Three days later, non-adherent cells were removed and fresh medium was replaced. A half volume of the culture medium was replaced every 3 days. The purity of hMDM culture *in vitro* was evaluated by staining with a human CD14 monoclonal antibody conjugated with R-phycoerythrin (Caltag Laboratories, CA, USA) as described previously [25].

### Primary neuron culture

Primary mouse neurons were isolated from cortices of early postnatal (P0) Balb/c mice as described previously [43], except that neurons were plated and maintained in NeuroCult™ SM1 media (Stemcell™ Technologies, Vancouver, Canada). In brief, pups were decapitated and the brain was collected, washed, and placed into the dissection media. Meninges and non-cortical forebrain tissues were removed with fine-point forceps. The cortex was collected and separated into a single-cell suspension by incubating in 20 U/mL papain solution (Sigma-Aldrich) for 10 minutes, followed by the addition of 100 U DNase I (Sigma-Aldrich) and incubation for 5 more minutes, gentle trituration with a fire-polished glass Pasteur pipette, and filtration through a 0.45-μm cell strainer. Cells were then resuspended in NeuroCult™ SM1 plating medium and 1 × 10^5^ cells were plated into wells of a 24-well plate coated with poly-D-lysine (molecular weight, 30 to 70 K; Sigma-Aldrich). A half volume of the culture medium was replaced with fresh NeuroCult™ SM1 maintenance medium every 3 days. Neurons were maintained at 37°C in 5% CO_2_ for 6 days before treatments.

### Transduction of human cell lines and primary hMDM

HTB-11 cells were transduced with lentiviral vectors and the transduction efficiency was evaluated following methods described previously [25]. Briefly, HTB-11 cells were sub-cultured at a density of 5 × 10^5^ in a T25 tissue culture flask 24 hours before transduction. For transduction, cell culture medium was removed and cells were washed twice with Dulbecco’s Phosphate-Buffered Saline (DPBS) (Corning Life Sciences) followed by addition of 0.5 mL vector stock (multiplicity of infection, MOI = 10) containing 8 μg/mL polybrene (Sigma-Aldrich), and incubated at 37°C in 5% CO_2_ for 2 hours. The vector suspension was removed and fresh growth medium was added. The medium was replaced 24 hours later and transduction efficiency was evaluated on day 3 post-transduction. The percentage of GFP^+^ cells was determined by calculating the number of GFP^+^ cells and total cells from randomly selected microscopic fields using an epi-fluorescence microscope (Nikon Eclipse TE2000-U). All experiments were performed in triplicate and a total of 5 random microscopic fields, each containing at least 100 cells, were counted for each test.

U937 cells were transduced using a spin-infection method. Approximately 1 × 10^5^ cells were resuspended in 100 μL of vector suspension (MOI = 100) in the presence of 8 μg/mL polybrene (Sigma-Aldrich) and plated into a 48-well plate. The plate was sealed and spun at 1,500 × *g* for 90 minutes at 32°C. Cells were washed with fresh medium and plated into a 12-well plate and cultured at 37°C in 5% CO_2_. A second-round transduction was performed the next day. The transduction efficiency was evaluated on day 8 post-transduction as described above.

hMDMs cultured in 12-well plates were infected with HR-Hutat2 vectors at the MOI of 10 or 50 in the presence of 8 μg/mL polybrene for 1.5 hours on days 7 and 8 *in vitro* (DIV 7 and DIV 8), respectively. The transduction efficiency was evaluated on day 8 post-transduction (DIV 16). All experiments were performed in triplicate. A total of five random microscopic fields were counted for each test.

### Western blotting

For western blot assay, cells were washed with DPBS three times, cultured in the serum-free medium, and harvested 2 days later. Cells were lysed with RIPA lysis buffer containing protease inhibitor cocktail (G-Biosiences, St. Louis, MO, USA). The equal volume of serum-free supernatants or the equal amount of total proteins in lysates from transduced or non-transduced cells, including HTB-11, U937, and hMDM cells, were mixed with 5× sodium dodecyl sulfate (SDS) sample buffer and loaded on 4% stacking/7.5% separating SDS-polyacrylamide gels (GibcoBRL, Grand Island, NY, USA). Following electrophoresis at 100 V for 1.5 hours, separated proteins were transferred onto a nitrocellulose membrane (NCM; GE Hybond ECL, Pittsburgh, PA, USA). The NMCs were saturated with 1% bovine serum albumin (BSA; Sigma-Aldrich) in Tris-buffered saline containing 0.05% Tween 20 (TBST; Sigma-Aldrich) for 1 hour at room temperature (RT), followed by incubation with rabbit-anti-human IgG_(H+L)_ (1:1,000 dilution) (Rockland, Gilbertsville, PA, USA) for 1 hour at RT. Following extensive washing with TBST, the NCM was incubated with horseradish peroxidase (HRP)-conjugated goat-anti-rabbit IgG at a dilution of 1:3,000 (Rockland) at RT for 1 hour, and then washed three more times with TBST prior to the exposure to a metal enhanced 3,3-diaminobenzidine tetrahydrochloride (DAB) substrate (PIERCE, Rockford, IL, USA) for identification of protein bands. Equal lane loading was assessed using a rabbit anti-β-actin antibody at a dilution of 1:1,000 (Rockland).

### Enzyme-linked immunosorbent assay (ELISA)

Human IgG ELISA was used to quantify the secreted Hutat2:Fc in the culture mediums from transduced HTB-11, U937, and hMDM. A 96-well plate was coated with a goat-anti-human IgG Fc capture antibody (Rockland) overnight at 4°C. The plate was then washed three times with TBST and blocked with TBS containing 1% BSA (Sigma-Aldrich) for 30 min at RT on an orbital shaker. After washing three times with TBST, the plate was incubated with diluted Hutat2:Fc containing supernatant samples for 1 hour and then incubated with a goat anti-human IgG Fc biotin-conjugated detection antibody (Rockland) for 1 hour. The plate was then washed and finally incubated with streptavidin-HRP (Rockland) for 30 min at RT. The presence of Hutat2:Fc protein was detected with tetramethylbenzidine (eBioscience, San Diego, CA, USA). The enzymatic reaction stopped by adding 50 μL of 1 M sulfuric acid. The optical density values were read at 450 nm with 570 nm as the reference wavelength using a microplate reader (Beckman Coulter AD340, Fullerton, CA, USA), and compared with a standard curve of human IgG protein (Sigma-Aldrich, cat# I2511). Finally, the IgG concentration (C_IgG_) was transformed to Hutat2:Fc concentration (C_Hutat2:Fc_) in according to the molecular weight (MW) ratio of Hutat2:Fc to IgG (C_Hutat2:Fc_ = C_IgG_ × MW_Hutat2:Fc_/MW_IgG_).

Endogenous IL1β, IL8, IL10, and TNF-α levels in the supernatants of hMDM and transduced hMDM were quantified with commercial ELISA kits (human IL1β, IL10, and TNF-α ELISA Ready-SET-Go! kit, eBioscience; human IL8 ELISA MAX™ kit, BioLegend, San Diego, CA, USA) following the manufacturers’ instructions.

### Dot-immunobinding assay (DIBA)

NCM strips were equilibrated in TBS and then air-dried; 200 ng of HIV-1 Tat_86_ (100 μg/mL) (NIH AIDS Reagents Program, Cat#2222) or Tat dilution buffer were spotted onto the NCM directly and allowed to air-dry for 30 min. After being blocked with 1% BSA in TBST, the loaded membranes were incubated with conditioned mediums collected from HR-Hutat2 transduced HTB-11, U937, and hMDM, or from HR-A3H5 transduced HTB-11 at 4°C overnight. Rabbit-anti-human IgG_(H+L)_ (1:1,000 dilution) (Rockland) and goat anti-rabbit IgG HRP-conjugated (1:3,000 dilution) (Rockland) were used before the exposure to a metal enhanced DAB substrate (PIERCE). Specific binding was visualized by the color deposition on the NCM. The Tat-loaded membrane incubated with rabbit anti-Tat serum (1:1,000 dilution) (NIH AIDS Reagents Program, Cat#705) followed by the incubation with HRP-conjugated goat anti-rabbit IgG (1:3,000 dilution) served as a positive control.

### Real-time PCR

Total mRNA was extracted from cell samples using High Pure RNA Isolation Kit (Roche, Germany) following the manufacturer’s instructions. Total RNA concentration was estimated from absorbance at 260 nm (A260; Beckman Coulter DU 800) and RNA quality was verified by electrophoresis on ethidium bromide-stained 1.5% agarose gels and by A260/A280 ratios >1.8.

Primers were designed with Primer-BLAST online (http://www.ncbi.nlm.nih.gov/tools/primer-blast/index.cgi?LINK_LOC=BlastHome) and/or express Designer™ module of express Profiler™ software (Beckman Coulter), and synthesized from Integrated DNA Technologies (Coralville, IA, USA). Specific primer pairs were used for the expression studies as follows: Hutat2: 5′-ACATCTGTGGTTCTTCCTTCTCCT-3′/5′-TCACTCCATATCACTCCCAGCCACTC-3′; EGFP: 5′-GGTGAGCAAGGGCGAGGAG-3′/5′-GCCGGTGGTGCAGATGAACT-3′; ACTB: 5′-AGGTGACACTATAGAATAGGCATCCTCACCCTGAAGTA-3′/5′-GTACGACTCACTATAGGGACAGAGGCGTACAGGGATAGC-3′. For the gene expression profiling analysis between transduced and non-transduced hMDM, in total, 15 primer pairs targeting pro-inflammatory cytokines genes, apoptosis-related genes, tumor-related genes, and cell signal transduction genes were used (Table 1). Three reference genes *ACTB*, *GK*, and *Ezrin* were used for these normalizations. Primer specificity was confirmed by capillary gel electrophoresis using GenomeLab™ GeXp gene analysis system (Beckman Coulter) and melt curve analysis. When capillary gel electrophoresis was performed, a universal sequence (Forward: 5′-AGGTGACACTATAGAATA-3′; Reverse: 5′-GTACGACTCACTATAGGGA-3′) was added to the 5′-end of each forward or reverse primer following the manufacturer’s instructions. cDNA was synthesized from 1 μg DNase-treated total RNA by using a Transcriptor First Strand cDNA Synthesis Kit (Roche, Germany) according to the manufacturer’s instructions.

**Table 1:** **Primer list for human monocyte-derived macrophage (hMDM)-related functional and regulatory gene expression analysis**

	**Gene name**	**Accession number [GenBank]**	**Description**	**Forward sequence (5′ → 3′)**	**Reverse sequence (5′ → 3′)**
1	*IL18*	NM_001562	Interleukin 18	TCAGACCTTCCAGATCGCTT	TGCCACAAAGTTGATGCAAT
2	*FAS*	NM_000043	Fas cell surface death receptor	ACTGTGACCCTTGCACCAAA	GCCACCCCAAGTTAGATCTGG
3	EZR*	X51521	Ezrin	GCCTAGAGGCTGACCGTATG	TGTGTATTCTGCAAGCTCCG
4	*P53*	NM_000546	Tumor protein p53	CTTCGAGATGTTCCGAGAGC	TTATGGCGGGAGGTAGACTG
5	*TLR1*	NM_003263	Toll-like receptor 1	GACTGCCAAATGGAACAGACA	AGGGCCTGGTACCCCTATTA
6	GK*	NM_203391	Glycerol kinase	CTACAATGCTGTGGTGTGGC	TCAAGGAGCCAACGAAGTTT
7	*IL1β*	NM_000576	Interleukin 1β	CCTCCAGGGACAGGATATGGA	CCAGCTGTAGAGTGGGCTTA
8	*IL8*	NM_000584	Interleukin 8; neutrophil chemotactic factor	CACCGGAAGGAACCATCTCA	TTGGGGTGGAAAGGTTTGGA
9	*IDO1*	NM_002164	Indoleamine 2,3-dioxygenase 1	TATTTGTCTGGCTGGAAAGGC	GGAGGAACTGAGCAGCATGTC
10	*NFKB2*	NM_001077494	Nuclear factor of kappa light polypeptide gene enhancer in B-cells 2 (p49/p100)	AGCAAGAGGCCAAAGAACTG	TGCTGTCTTGTCCATTCGAG
11	*IFNGR2*	NM_005534	Interferon gamma receptor 2	CTGATCTCCGTGGGAACATT	TCCTTTGGTGAGCTGTCCTT
12	*STAT1*	NM_007315	Signal transducer and activator of transcription 1	CTGAGGAGTTTGACGAGGTGT	CTATCAACAGGTTGCAGCGAA
13	*CCR5*	NM_000579	Chemokine (C-C motif) receptor 5	GGGATAGCACTGAGCAAAGC	TCTGAAATACGGAGGCTGGT
14	*CCL2*	NM_002982	Chemokine (C-C motif) ligand 2; monocyte chemotactic protein-1 (MCP-1)	GCCTCCAGCATGAAAGTCTC	AGATCTCCTTGGCCACAATG
15	*CASP3*	NM_004346	Caspase3, apoptosis-related cysteine peptidase	AGCGAATCAATGGACTCTGG	AACATCACGCATCAATTCCA
16	ACTB*	NM_001101	Beta-actin	GGCATCCTCACCCTGAAGTA	CAGAGGCGTACAGGGATAGC
17	*IL10*	NM_000572	Interleukin 10	CGGCGCTGTCATCGATTTCT	GTTTCTCAAGGGGCTGGGTC
18	*TNF-α*	NM_000594	Tumor necrosis factor-α	CAGAGGGAAGAGTTCCCCAG	CCTTGGTCTGGTAGGAGACG

*Reference gene.

Real-time PCR was performed with i-Cycler iQ5 Real-time Detection System (Bio-Rad Laboratories Inc., Hercules, CA, USA). Each sample was amplified in triplicate using 96-well optical plates (Bio-Rad Laboratories Inc.) in a 20 μL reaction volume using 2 μL cDNA (10 ng/μL), 10 μL FastStart Universal SYBR Green Master (Roche), and 200 nM of the appropriate forward and reverse primers. Real-time cycling conditions were as follows: initial denaturation at 95°C for 10 min, followed by 40 cycles of denaturation at 95°C for 15 sec and annealing at 60°C for 60 sec, and, finally, melt curve analysis. Template-minus and reverse transcriptase-minus negative controls were run for each plate and each sample, respectively. Fold change of gene expression was determined using the arithmetic comparative method (2^-ΔΔCt^). Three batches of transduced or mock control cells were tested in all experiments. All real-time PCR tests were done in triplicates.

### Fluorescent immunocytochemistry

Cells were fixed with 4% paraformaldehyde for 10 min and blocking was performed by incubating for 15 min with 1% BSA in PBS containing 0.1% Triton X-100 (Sigma-Aldrich). For human IgG Fc staining, cells were incubated with primary antibody, rabbit anti-human IgG Fc at a dilution of 1:100, and a secondary antibody goat-anti-rabbit IgG conjugated with tetraethyl rhodamine isothiocyanate (TRITC) at a dilution of 1:200 (Rockland Immunochemical). For mouse neuron staining, cells were incubated with a rabbit anti-microtubule-associated protein 2 (MAP2) antibody (1:200 dilution) (Millipore, Billerica, MA, USA), and then incubated with a goat anti-rabbit IgG TRITC conjugated antibody (1:200 dilution) (Jackson ImmunoResearch Laboratories, West Grove, PA, USA). For HIV-1 p24 staining, after fixing and blocking, hMDM were incubated with an anti-HIV-1 p24 monoclonal antibody (1:500 dilution) (NIH AIDS Reagents Program, Cat#530) followed by incubation with goat anti-mouse IgG conjugated to biotin (1:200 dilution) (Vector labs, Burlingame, CA, USA), and with streptavidin-TRITC (1:200 dilution) (Jackson ImmunoResearch Laboratories) subsequently. For CD14 staining, cells were not fixed and permeabilized. After three washings with PBS, hMDM were incubated with a human CD14 monoclonal antibody conjugated with R-phycoerythrin (1:100 dilution) (Caltag Laboratories, CA, USA). To stain nuclei, cells were incubated with 4′,6-diamidino-2-phenylindole (DAPI) (Sigma-Aldrich) for 5 min. All the incubations were followed by extensive washing with PBS. Negative controls consisted of pre-incubation with 1% BSA in PBS containing 0.1% Triton X-100 and omission of the primary antibody followed by corresponding secondary antibody. To detect apoptosis in neurons, a terminal dexoynucleotidyl transferase-mediated dUTP nick end labeling (TUNEL) assay using MEBSTAIN Apoptosis TUNEL Kit II (MBL, Woburn, MA, USA) was performed according to the manufacturer’s instructions. Immunofluorescent images were obtained with an inverted epi-microscope (Nikon Eclipse TE2000-U) using a numerical aperture lens (0.30 or 0.45) and a digital camera attachment. The pictures were overlaid using ImageJ software (Version 1.48, National Institutes of Health, USA).

### MTT assay

HTB-11 cells at the exponential growth phase were seeded into 96-well plates at 1 × 10^4^ cells/well in 100 μL and cultured for 48 hours. Twenty milliliters of MTT solution (5 mg/mL) (Sigma-Aldrich) was added to the 100 μL of medium in each well, and the plate incubated at 37°C for 4 hours. The solution was removed, followed by the addition of 100 μL/well of dimethylsulfoxide (Amresco, Solon, OH, USA) to solubilize the purple formazan crystals produced. Absorbance in each well was measured at 570 nm using a 96-well plate reader (Beckman Coulter AD340). To evaluate the neuroprotective effects of the Hutat2:Fc, HTB-11 cells were treated with HIV-1 Tat_86_ (500 nM), Tat_86_, plus the conditioned medium from HR-Hutat2 transduced HTB-11, U937, or hMDM at a dilution of 1:5. Treatment with Tat_86_ plus anti-Tat antibody was used as a positive control, while Tat_86_ plus the conditioned mediums from the HR-A3H5 transduced HTB-11 was used as a negative control. Seventy-two hours later, an MTT assay was performed as noted above. In some experiments, the vector HR-Hutat2 transduced HTB-11 cells were treated with HIV-1 Tat_8_6 (500 nM) for 72 hours and an MTT assay was performed. The vector HR-A3H5-transduced HTB-11 treated with HIV-1 Tat_86_ was used as a negative control. All experiments were performed in quadruplicate.

### Primary neuron protection assay

For this experiment, all the tested conditioned mediums were FBS-free to avoid possible stimulation of astrocyte growth, and the conditioned mediums from the transduced hMDM on day 9 post-transduction were tested as representative samples, since the mediums contained the highest level of Hutat2:Fc as compared to the supernatants harvested on the other days. Mouse primary neurons cultured in 24-well plates were treated with HIV-1 Tat_86_ (500 nM) alone, or Tat adding with the conditioned mediums from HR-Hutat2 transduced hMDM or HTB-11 (1:5 dilution) on DIV 6 for 3 days. Treatments with Tat_86_ plus anti-Tat monoclonal antibody (NIH AIDS Reagents Program, Cat#7377) was used as a positive control while Tat_86_ plus the conditioned mediums from the HR-A3H5 transduced HTB-11 was used as a negative control, respectively. Three days later (DIV 9), cells were fixed with 4% paraformaldehyde and counterstained with anti-MAP2 (neuron) followed by TUNEL (apoptosis) labeling, and DAPI (nuclei) staining as described above. Fields were chosen randomly, and at least five images from five random fields were acquired with an epi-fluorescence microscope (Nikon Eclipse TE2000-U) from each of three independent experiments. In normal neuron culture, there were some TUNEL-positive cells. It was reported that these represented non-neuronal dividing cells that were undergoing cell death and apoptotic neurons from the preparation procedure [43]. Note that around these structures intact cell bodies were not observed when the images were overlaid together. Therefore, in this neuron survival evaluation, only the neurons which had intact cell bodies (red) and nuclei (blue), yet were resistant to TUNEL labeling (green), were calculated as survivals. The number of surviving neurons and total neuron numbers were counted manually. The ratio of living neurons in normal neuron culture was arbitrarily defined as 100% neuron survival rate. The relative neuron survival rate (%) was expressed as a percentage relative to the untreated control neurons. Each value is the mean obtained from five random microscopic fields of three independent experiments using a 20 × objective.

### HIV-1 challenge

HIV-1_Ba-L_ strain (R5) was obtained from the NIH AIDS Reagent Program (Cat#510). Human MDM were isolated and transduced with HR-Hutat2 vectors on DIV 7 and DIV 8. Six-days later, non-transduced hMDM, transduced hMDM, non-transduced hMDM with anti-HIV-1 Tat monoclonal antibody (1:100 dilution), or the conditioned medium from transduced hMDM (1:2 dilution) were incubated with cell-free HIV-1_Ba-L_ (final concentration of p24 7.8 ng/mL) at 37°C for 2 hours, respectively. Cells were washed three times and fresh medium was added. Half volumes of the culture supernatants were collected and replaced with fresh medium every 3 days for a total of 24 days. Anti-HIV-1 Tat or the conditioned medium from transduced hMDM were supplemented to the appropriate wells when medium was replaced. Viral replication was gauged for p24 levels in the culture supernatants using a commercial HIV-1 p24 ELISA kit (Beckman Coulter) in accordance with the manufacturer’s instructions. The blood from three donors was used in this test and triple independent experiments were performed.

### Statistical analysis

Statistical analyses were performed by running the SPSS Version 16.0 for Windows package. Data were reported in the text as means ± standard error means (s.e.m). Student’s *t*-test and χ^2^ test were used to determine the statistical significance of independent data, appropriately. One-way analysis of variance (ANOVA) followed by Tukey’s multiple comparison *post hoc* test was used to analyze studies with three or more experimental groups. Comparisons of each group with the control used Dunnett test. The *P* values were two-tailed and a *P* value less than 0.05 was considered to be significant.

## Results

### Evaluation of the gene transfer efficiency and the stable expression of anti-HIV-1 Tat Hutat2:Fc in human neuronal cell line HTB-11, monocytic cell line U937, and primary hMDM

**Figure 1:**
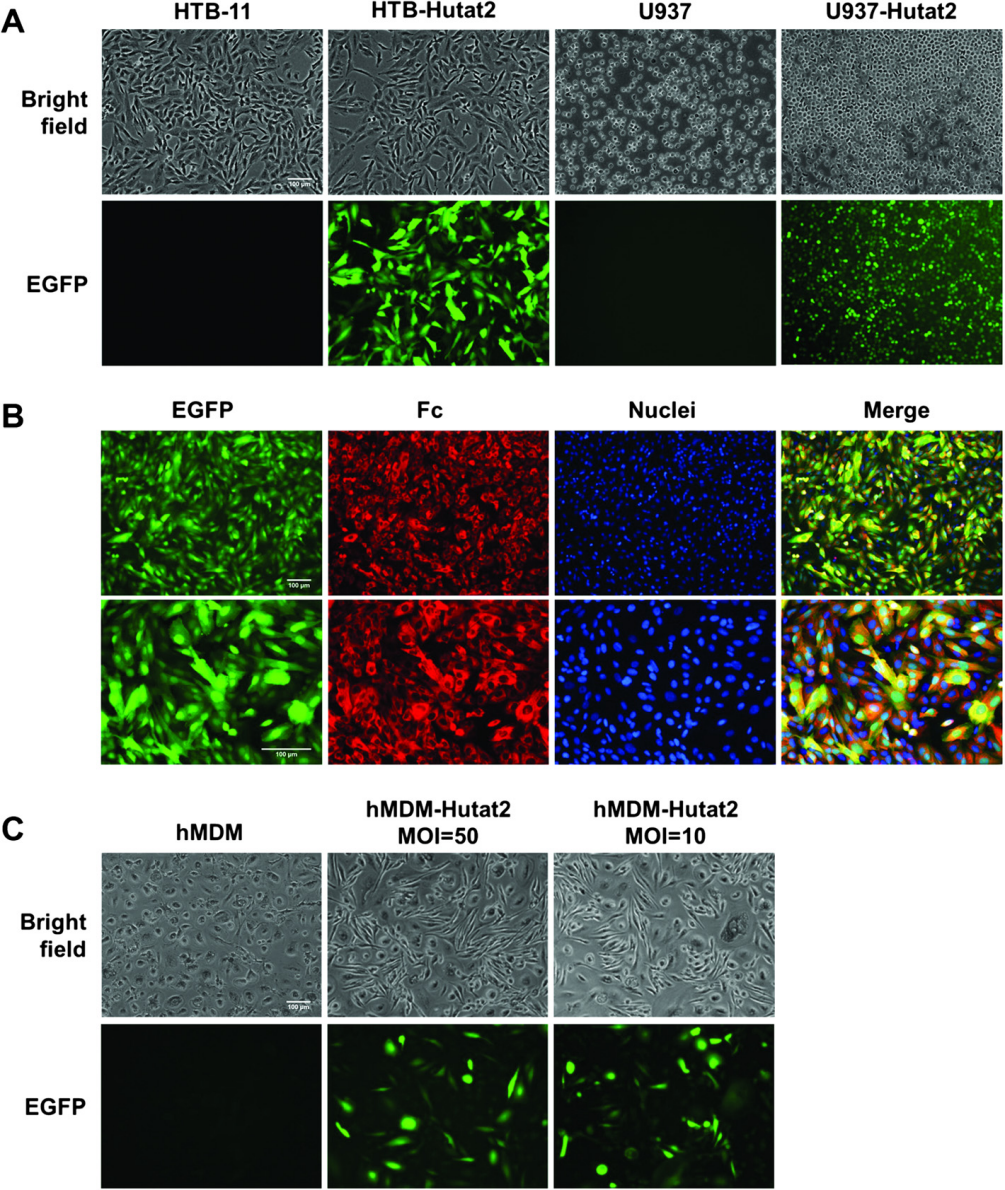
**Transduction of human cell lines HTB-11 and U937 as well as primary hMDM by lentiviral vectors HR-Hutat2 expressing anti-HIV-1 Hutat2:Fc and EGFP.** HTB-11 cells (5 × 10^5^) were transduced in a T25 flask in the presence of 8 μg/mL polybrene for 2 h (multiplicity of infection, MOI = 10). U937 cells (1 × 10^5^) were transduced twice by spin-infection at 1,500 × *g* for 90 minutes (MOI = 100). Human MDM were infected with HR-Hutat2 vectors (MOI = 50 or MOI = 10) for 1.5 h on days 7 and 8 *in vitro* (DIV 7 and DIV 8), respectively. The transduction efficiencies were evaluated by calculating the percentage of GFP^+^ cells from five randomly selected microscopic fields under a fluorescence microscope on day 3 post-transduction for HTB-11, as well as on day 8 post-transduction for U937 and hMDM, respectively. HTB-11, Non-transduced HTB-11 cells; HTB-Hutat2, HR-Hutat2 transduced HTB-11 cells; U937, Non-transduced U937 cells; U937-Hutat2, HR-Hutat2 transduced U937 cells; EGFP, Enhanced green fluorescent protein; hMDM-Hutat2 MOI = 50, HR-Hutat2 transduced hMDM at the MOI of 50; hMDM-Hutat2 MOI = 10, HR-Hutat2 transduced hMDM at the MOI of 10. **(A)** Expression of EGFP in HR-Hutat2 transduced HTB-11 and U937 cells. **(B)** Co-location of the Hutat2:Fc and EGFP expression in HR-Hutat2 transduced HTB-11. Nuclei were counterstained with DAPI (blue). The Hutat2:Fc proteins (red) were expressed in the cytoplasm while EGFP proteins (green) were expressed both in the nuclei and cytoplasm. **(C)** Expression of EGFP in transduced hMDM. Fluorescently-labeled cells were visualized with an epi-microscope (Nikon Eclipse TE2000-U) using a numerical aperture lens (0.30 or 0.45) and a digital camera attachment. The pictures were overlaid using ImageJ software (Version 1.48, National Institutes of Health, USA). Data represent means ± s.e.m. of three independent experiments. Scale bar = 100 μm.

The efficiency of lentiviral vector-mediated gene transfer was evaluated initially in human neuronal and monocytic cell lines. Human neuroblastoma cell line HTB-11 and monocytic cell line U937 were transduced with lentiviral vectors HR-Hutat2 at a MOI of 10 and 100, respectively. Under the established experimental conditions, transduction efficiencies were calculated to be 98.5% ± 0.8% for HTB-11 cells and 95.4% ± 2.5% for U937 cells (Figure 1A). Furthermore, the expression of the integrated genes was confirmed by examining transduced HTB-11 for the Fc expression using immunofluorescent staining with an anti-human IgG Fc specific antibody. EGFP proteins were expressed in both the nuclei and cytoplasm, whereas Hutat2:Fc was predominately distributed in the cytoplasm (Figure 1B). HTB-11 cells were also transduced with control vectors HR-A3H5 containing a construct encoding the anti-Epstein-Barr virus latent membrane protein 1 scFv A3H5 fused to Fc. The transduction efficiency was as high as that obtained from HR-Hutat2 transduced HTB-11 cells (data not shown).

Next, we tested whether the vector HR-Hutat2 could successfully transduce non-dividing primary hMDMs. The purity of the cultured hMDMs was proved to be >98% by CD14^+^ immunofluorescent staining on DIV 6 (Additional file 2). hMDMs were infected with the concentrated HR-Hutat2 stock (MOI = 50) or unconcentrated stock (MOI = 10) on DIV 7 and DIV 8. The transduction efficiencies were approximately 53.3% and 47.6%, respectively (Figure 1C). There were no significant differences in the transduction efficiency between the two MOI groups (*P* >0.05).

**Figure 2:**
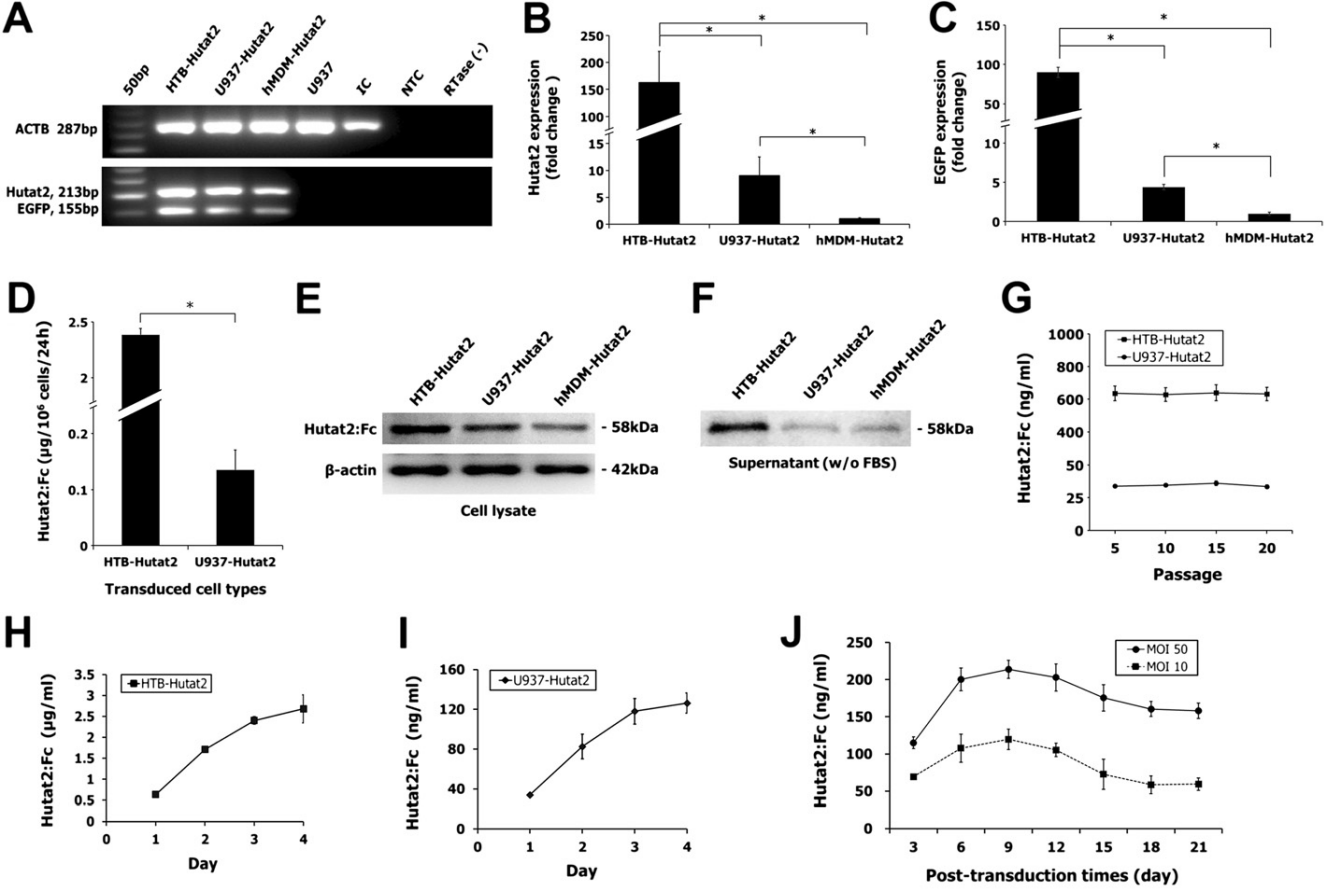
**Relative gene expression levels of the** ***Hutat2:Fc*** **and** ***EGFP*** **genes in transduced cells and quantification of Hutat2:Fc in conditioned mediums.** **(A)** Detection of *Hutat2* and *EGFP* mRNA in HR-Hutat2 transduced cells by a RT-PCR qualitative analysis. HTB-Hutat2, HR-Hutat2 transduced HTB-11 RNA; U937-Hutat2, HR-Hutat2 transduced U937 RNA; hMDM-Hutat2, HR-Hutat2 transduced hMDM RNA; U937, non-transduced U937 RNA; IC, Internal control RNA from K562 cell line; NTC, No template control; RTase (−), RTase negative control. **(B and C)** Quantitative real-time PCR analysis of *Hutat2* and *EGFP* gene expression levels in transduced HTB-11 and U937 cells compared with that in transduced hMDM (*P <0.01). **(D)** Comparison of Hutat2:Fc secretion level between transduced HTB-11 and U937 within 24 hours (*P <0.01); 1 × 10^6^ cells were plated into a T-75 flask and the mediums were collected 24 hours later. Hutat2:Fc was quantified by a human IgG ELISA method. **(E and F)** Detection of Hutat2:Fc proteins in cell lysate and supernatant of transduced cells by Western blotting. **(G)** Detection of stable secretion of Hutat2:Fc in conditioned mediums from HR-Hutat2 transduced HTB-11 (HTB-Hutat2) and U937 cells (U937-Hutat2). Cells were passaged totally 20 times and an ELISA assay was performed every fifth passage. **(H and I)** The accumulation of Hutat2:Fc in mediums from transduced HTB-11 and U937 cells; 1 × 10^6^ cells were plated into a T-75 flask and the mediums were collected every 24 hours for 4 days. **(J)** Kinetics of Hutat2:Fc levels in cell culture supernatants of transduced hMDM at different MOI after transduction. The levels of secreted Hutat2:Fc were peak on day 9 post-transduction. The concentrations of Hutat2:Fc were higher at MOI 50 than at MOI 10 in mediums of transduced hMDM at each time point (*P* <0.01). Results shown represent mean values from three independent experiments. Error bars denote the s.e.m.

Furthermore, the transcriptional profiling for the integrated *Hutat2* and *EGFP* genes in transduced HTB-11, U937, and hMDM were examined by RT-PCR analysis (Figure 2A) and confirmed by a real-time PCR test. The expression of *Hutat2* and *EGFP* genes in transduced cells was normalized with three reference genes (*ACTB*, *GK*, and *Ezrin*) and compared with transduced hMDM. The expression levels of the *Hutat2* gene in transduced HTB-11 and transduced U937 were 162.5- and 9.0-fold higher than that in transduced hMDM, respectively, while the expression level of the *Hutat2* gene in transduced HTB-11 was 18.1-fold higher than that in transduced U937 (Figure 2B). In addition, the expression levels of *EGFP* in transduced HTB-11 and U937 cells were 89.7- and 4.4-fold higher than that determined in transduced hMDM, respectively (Figure 2C). The difference in the gene expression between different transduced cells was further confirmed by an ELISA quantification of Hutat2:Fc secreted in the supernatants of transduced HTB-11 and U937 cells. It was shown that the secretion of Hutat2:Fc in the supernatants of transduced HTB-11 was 17.1-fold higher than that in the supernatants of transduced U937 cells (2.39 ± 0.11 μg/10^6^ cells/24 h compared with 0.14 ± 0.04 μg/10^6^ cells/24 h, *P* <0.01) (Figure 2D).

The expression and secretion of Hutat2:Fc in the transduced HTB-11, U937, and hMDM cells were confirmed by Western blotting. To assess the production of Hutat2:Fc extracellularly and intracellularly, cell culture supernatants containing no FBS and cell lysates from the above transduced cells were collected or extracted. Vector-transduced cells expressed Hutat2:Fc both within cells (cell lysate) and in secreting form (culture supernatant) (Figure 2E,F). The molecular weight of Hutat2:Fc fusion protein is approximate 58 kDa.

To quantify the stability of Hutat2:Fc expression and secretion, transduced HTB-11 and U937 cells were sub-cultured *in vitro* for up to 20 passages. The concentration of Hutat2:Fc in the conditioned medium was assessed using an ELISA method at every fifth passage. Our data showed the levels of Hutat2:Fc in the conditioned mediums were stable over the course of 20 cell passages in HR-Hutat2 transduced cells (Figure 2G). The percentage of GFP^+^ cells in these transduced cell populations was also found to be stable through the course of the 20 passages (data not shown). In addition, the secreted Hutat2:Fc could be accumulated in the conditioned mediums of transduced HTB-11 and U937 during a 4-day examination (Figure 2H,I). The concentration increased exponentially with time and reached to plateau on day 4 (2.68 ± 0.33 μg/mL for HTB-Hutat2 and 126.16 ± 10.12 ng/mL for U937-Hutat2).

The levels of secreted Hutat2:Fc in cell culture supernatants of transduced hMDM were peak on day 9 post-transduction (DIV 17) in both the MOI 50 group (213.83 ± 12.03 ng/mL) and MOI 10 group (119.66 ± 13.64 ng/mL), and then gradually fell to 158.06 ± 10.41 ng/mL and 59.45 ± 8.36 ng/ml in these two groups on day 21 (DIV 29), respectively (Figure 2J). The Hutat2:Fc secreted into the cell culture mediums could be detected as early as day 3 post-transduction, expressed much earlier than the expression of EGFP, which became visibly apparent on day 8 post-transduction. These findings as well as the gene expression profiling indicated that the expression of genes co-expressed through an IRES element was weaker than the promoter-proximal gene(s) [44]. Transduced hMDM were maintained in good condition for up to 30 days *in vitro*.

### Specific binding of expressed Hutat2:Fc to HIV-1 Tat

**Figure 3:**
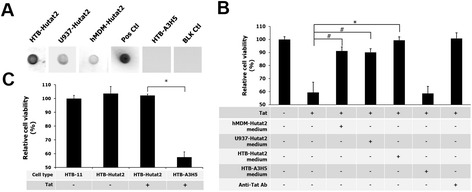
**Evaluation of the biological binding function of Hutat2:Fc and protective effects of Hutat2:Fc against HIV-1 Tat**_**86**_ **-mediated toxicity in HTB-11 cells.** **(A)** Specific binding of Hutat2:Fc to HIV-1 Tat. HIV-1 Tat_86_ (Clade B) loaded nitrocellular membranes (NCM) were incubated with cell culture supernatants collected from HR-Hutat2-transduced HTB-11 (HTB-Hutat2), U937 (U937-Hutat2), or hMDM (hMDM-Hutat2) at 4°C overnight followed by incubation with rabbit anti-human IgG_(H+L)_ and goat anti-rabbit IgG HRP conjugated antibodies. Specific binding was visualized by the color deposition on the NCM. The Tat_86_-loaded membrane incubated with rabbit anti-Tat serum served as a positive control (Pos Ctl) while incubated with cell culture supernatant from HR-A3H5 transduced HTB-11 served as a negative control (HTB-A3H5). The NCM loaded with Tat dilution buffer was used as a blank control (BLK Ctl). **(B)** Functional antagonization of Hutat2:Fc against HIV-1 Tat_86_-induced toxicity in HTB-11 cells by an MTT assay. The OD_570_ value of untreated HTB-11 cells was arbitrarily defined as 100% cell viability. The relative cell viability (%) was expressed as a percentage relative to the untreated control cells. The cell viability was significantly higher for the cells treated with the conditioned mediums from transduced cells releasing Hutat:Fc when compared to the cultures that received Tat_86_ (500 nM) alone (*P <0.01 for HTB-Hutat2 medium; ^#^ *P* <0.05 for U937-Hutat2 medium, and hMDM-Hutat2 medium). **(C)** Protection of HR-Hutat2 transduction against Tat_86_-induced toxicity by an MTT assay. No significant difference of cell viability was detected between normal and vector HR-Hutat2 transduced HTB-11 cells (HTB-Hutat2) (*P* >0.05). However, the cell viability of HTB-11 transduced with the vector HR-Hutat2 was significantly higher than that of HTB-A3H5 in the presence of HIV-1 Tat_86_ (500 nM) (*P <0.01). All experiments were performed in quadruplicate. Error bars denote the s.e.m.

After confirming the stable expression of Hutat2:Fc, an immunoblot assay was employed to assess the specific binding ability of secreted Hutat2:Fc to HIV-1 Tat. Recombinant HIV-1 Tat_86_ (Clade B) was diluted and blotted onto a NCM with the dilution buffer included as a blank control. The conditioned medium from HR-A3H5 transduced HTB-11 served as a negative control and anti-HIV-1 Tat serum served as a positive control. The conditioned mediums containing anti-HIV-1 Tat Hutat2:Fc from transduced HTB-11 and U937 cells as well as hMDM bound specifically to HIV-1 Tat_86_ while no binding was detected to neither the blank control nor the secreted A3H5:Fc control (Figure 3A). In addition, to confirm that the Hutat2:Fc was able to bind the unaggregated form of Tat, Tat_86_ was separated by SDS-PAGE electrophoresis and Western blot assay was performed using the conditioned medium from transduced cells as primary antibodies. In accordance with the DIBA results, Hutat2:Fc from HR-Hutat2 transduced cells could specifically bind to Tat_86_ (14 kDa), whereas A3H5:Fc from HR-A3H5 transduced HTB-11 could not (Additional file 3). These tests demonstrate that the secreted Hutat2:Fc is able to bind specifically and sufficiently to HIV Tat_86_ as a fully-functional HIV-1 Tat antibody *in vitro*, as designed.

### Protection of Hutat2:Fc against HIV-1 Tat-mediated neurotoxicity

The next important step was to determine whether binding of anti-HIV-1 Tat Hutat2:Fc to HIV-1 Tat_86_ can successfully neutralize the neurotoxic properties of Tat_86_. The ability of Hutat2:Fc to antagonize the toxicity of HIV-1 Tat_86_ was assessed by using an MTT assay to determine if the secreted Hutat2:Fc or vector transduction was able to protect HTB-11 cells against the neurotoxic impact of HIV-1 Tat_86_. When exposed to Tat_86_ (500 nM), normal HTB-11 cells exhibited a reduced cellular viability (59.4 ± 7.8%). Comparatively, HTB-11 cells exposed to Tat_86_ in the presence of the conditioned mediums from HR-Hutat2 vector-transduced HTB-11, U937, or hMDM were protected from cellular cytotoxicity (cell viability was 99.4 ± 2.6%, 90.1 ± 2.8%, and 91.1 ± 3.1%, respectively; Figure 3B). The slightly lower level of cyto-protective effects of the conditioned medium from the transduced hMDM compared to that from the transduced HTB-11 was due to the lower concentration of Hutat2:Fc in the conditioned medium. Furthermore, when exposed to Tat_86_, HR-Hutat2 transduced HTB-11 cells also showed a dramatically increase in cell viability of 102.1 ± 1.1% in comparison to HR-A3H5-transduced HTB-11 cells, which only had a viability of 57.5 ± 3.8%. The viability of HR-Hutat2- transduced HTB-11, either exposed to HIV-1 Tat or not, was comparable to the normal HTB-11 control (Figure 3C). These data indicated that both HR-Hutat2-transduced HTB-11 itself and the Hutat2:Fc proteins in the supernatants significantly mediated the cytoprotective effects. Taken together, these data reflect the ability of Hutat2:Fc to neutralize the biological activity of Tat_86_.

**Figure 4:**
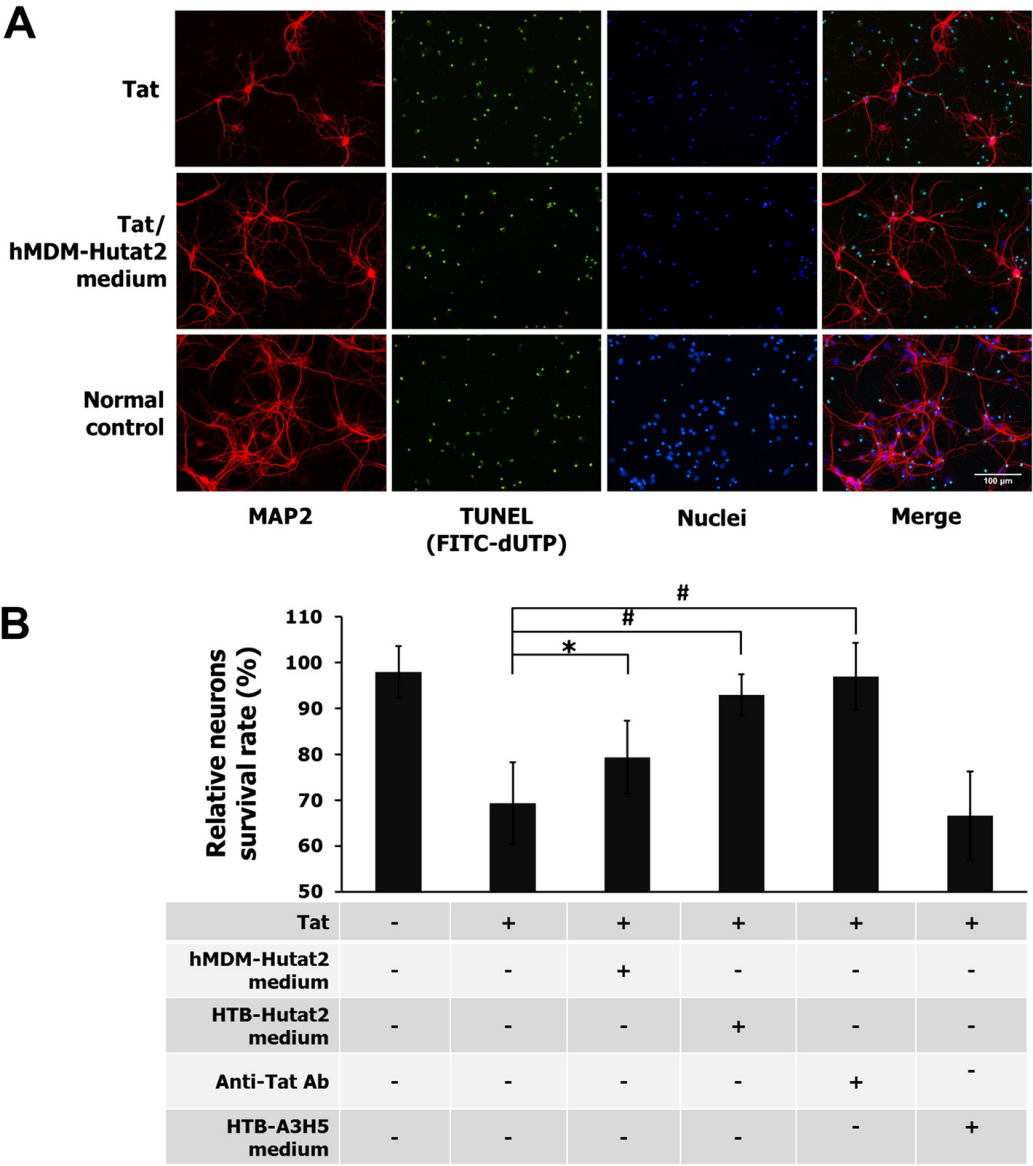
**Protection of the conditioned medium containing Hutat2:Fc against HIV-1 Tat**_**86**_ **-mediated neurotoxicity in primary mouse neurons.** Mouse cortical neurons cultured in 24-well plates were treated with HIV-1 Tat_86_ (Clade B, 500 nM) alone, or Tat with conditioned mediums from HR-Hutat2-transduced hMDM or HTB-11 (1:5 dilution) on day 6 *in vitro* (DIV 6) for 3 days. Treatment with Tat plus anti-Tat monoclonal antibody was used as a positive control, while Tat plus the conditioned medium from HR-A3H5 transduced HTB-11 was used as a negative control, respectively. **(A)** Representative images of primary mouse cortical neurons which were treated with HIV-1 Tat_86_ or Tat_86_ plus the conditioned medium from HR-Hutat2-transduced hMDM. Cells were counterstained with anti-MAP2 (MAP2), FITC-dUTP (TUNEL), and DAPI (Nuclei). Images of MAP2, TUNEL, and Nuclei were merged together (Merge). The survived neurons were the cells which were positive for MAP2 and DAPI but negative for TUNEL staining. Tat, Neurons treated with HIV-1 Tat_86_ alone; Tat/hMDM-Hutat2 medium, Neurons treated with HIV-1 Tat_86_ plus the conditioned medium of transduced hMDM; Normal control, Untreated neurons. Images were acquired as described in Figure 1. **(B)** Comparison of relative rates of neuron survival after treatment. The neuron survival rate of untreated neurons was defined as 100%. The relative neuron survival rate was increased by about 10% by adding Hutat2:Fc containing medium from transduced hMDM (*P <0.05 vs. treatment with Tat alone). However, the rate was still lower than normal neurons, neurons treated with Tat_86_ plus HTB-Hutat2 medium, and Tat_86_ plus anti-Tat antibody (^#^ *P* <0.01). Each value is the mean obtained from five random fields of three independent experiments using a 20× objective. Error bars denote the s.e.m. Scale bar = 100 μm.

In addition, these protective effects of Hutat2:Fc in the conditioned mediums were further evaluated using primary cultures of mouse neurons. Early postnatal (P0) Balb/c mouse neurons from cortex were isolated and cultured for 6 DIVs. The purity of the cultures were >95% neurons proved by MAP2 and glial fibrillary acidic protein immunocytochemistry staining (data not shown). The representative images of normal neurons and neurons treated with Tat_86_ or Tat_86_ plus Hutat2:Fc containing mediums from the transduced hMDM are shown in Figure 4A. Tat-treated mouse neurons showed increased numbers of cell apoptosis (Figure 4A TUNEL panel), loss of dendritic arbor, as well as a shorter dendrite length (Figure 4A; MAP2 panel). The relative rate of neuron survival was similar among normal neurons, neurons treated with Tat_86_ plus conditioned medium from HTB-Hutat2 (93.0 ± 4.5%), and neurons treated with Tat_86_ plus anti-Tat antibody (97.0 ± 7.2%). Compared with Tat exposure alone, the relative rate of neuron survival was increased by 10%, from 69.3 ± 8.9% to 79.4 ± 7.9% in the presence of conditioned medium from HR-Hutat2-transduced hMDM (*P* <0.05). However, the neuron survival rates were not significantly changed when adding HTB-A3H5 medium (66.6 ± 9.6% versus 69.3 ± 8.9%, *P* >0.05; Figure 4B). These results indicate that Hutat2:Fc released from transduced hMDM and HTB-11 could neutralize HIV-1 Tat_86_-induced neurotoxicity as an anti-Tat antibody *in vitro*, whereas A3H5:Fc released from HTB-A3H5 control does not have that biological effect. In comparison, the protective level of Hutat2:Fc from the conditioned medium of transduced hMDM was lower than that obtained from the use of transduced HTB-11 medium and the commercial anti-Tat antibody.

### Transduced hMDM culture and culture medium resist challenge with infectious HIV-1

To determine if HR-Hutat2-mediated transduction of hMDM could inhibit virus infection, both transduced and normal hMDM control were exposed to full-length infectious HIV-1_Ba-L_.

**Figure 5:**
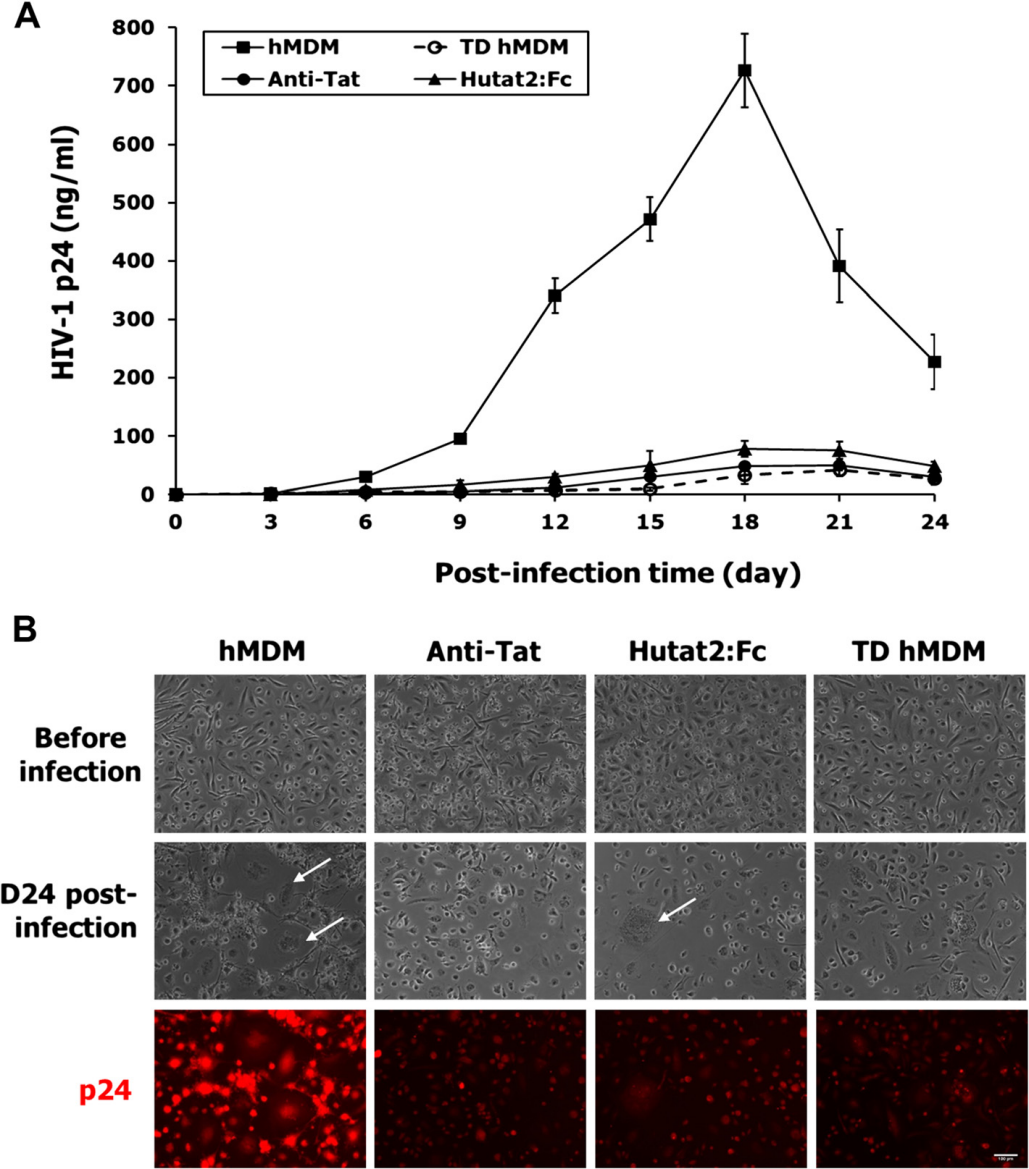
**Reducing of HIV-1 replication by lentivirus-mediated expression of Hutat2:Fc in primary hMDM.** **(A)** Kinetics of HIV-1_Ba−L_ replications (HIV-1 p24 levels). The data showed a significant reduction of HIV-1 replication in both the TD-hMDM and Hutat2:Fc culture groups as compared to hMDM (*P* <0.01), but no statistical difference among TD-hMDM, Hutat2:Fc, and Anti-Tat groups (*P* >0.05). **(B)** Lentiviral vectors HR-Hutat2 transduction suppresses HIV-1 cytopathicity and the expression of p24 in hMDM cultures. Normal hMDM and HR-Hutat2 transduced hMDM were exposed to HIV-1_Ba-L_, and examined before and on day 24 post-viral infection using a 10× objective. It can be readily appreciated that either HR-Hutat2 transduction or Hutat2:Fc strongly suppressed HIV-1-mediated cytopathic effects, resulting in a reduction in the number of giant cells in the culture. In addition, HIV-1 p24 immunofluorescent staining showed that HR-Hutat2 transduction and Hutat2:Fc reduced the expression of HIV-1 p24 intracellularly. Images were acquired as described in Figure 1. hMDM, Normal hMDM; TD-hMDM, HR-Hutat2 transduced hMDM; Anti-Tat, Non-transduced hMDM treated with anti-HIV-1 Tat antibody; Hutat2,Fc, Normal hMDM treated with conditioned medium from HR-Hutat2 transduced hMDM; D24 post-infection, Day 24 post-HIV-1-infection; p24, HIV-1 p24 immunofluorescent staining; White arrow, HIV-1-induced cytopathic effect. The blood of three donors was used in this assay. Results represent mean values from triple independent experiments and error bars denote the s.e.m. Scale bar = 100 μm.

hMDM was transduced with HR-Hutat2 on DIV 7 and DIV 8 and cultured for 6 days, then normal hMDM, HR-Hutat2-transduced hMDM, and hMDM supplemented with anti-HIV-1 Tat or with the conditioned medium from HR-Hutat2-transduced hMDM were infected with HIV-1_Ba-L_, respectively. The level of HIV-1 p24 production in these cultures was quantified by an ELISA assay (Figure 5A). HIV-1_Ba-L_ replication (p24 level) was detected in the control hMDM shortly after virus inoculation (day 3) and gradually increased with post-infection time, reaching the peak level by day 18 post-infection. The level of viral production dramatically suppressed (by 9- to 16-fold) in transduced hMDM-Hutat2 and normal hMDM supplemented with hMDM-Hutat2-conditioned medium or with anti-HIV-1 Tat antibody as compared to normal hMDM cultures (Figure 5A). These results suggest that the lentiviral vector-mediated Hutat2:Fc gene transfer conferred a significant degree of protection against wild-type HIV-1 infection in primary hMDM (*P* <0.01). In addition, the secreted Hutat2:Fc from transduced hMDM can suppress HIV-1_Ba-L_ propagation as an anti-HIV-1 Tat antibody. In agreement with this, an HIV-1-induced cytopathic effect in non-transduced hMDM was evident by the presence of abnormally large cells, multinucleated cells, and debris resulting from late stages of cell death. As a comparison, only very modest levels of HIV-1-induced cytopathic effects were observed in the transduced cultures or non-transduced culture supplemented with Hutat2:Fc conditioned medium (Figure 5B). Furthermore, although almost all of hMDM were infected by HIV-1_Ba-L_ after a 24-day culture period, the fluorescent signals of p24 staining in transduced hMDM or in normal hMDM treated with hMDM-Hutat2 conditioned medium were much weaker as compared to hMDM control (Figure 5B; p24 panel). These findings illustrate that although Hutat2:Fc is unable to completely block the cells from infection by HIV, lentiviral vector HR-Hutat2-transduced hMDM (intracellular Hutat2:Fc) and the Hutat2:Fc secreted from vector-transduced hMDM (extracellular Hutat2:Fc) are able to suppress HIV-1 replication and the spread of viral infection in macrophages.

### Potential adverse impacts

A vital component of gene therapy is to ensure that neither the method of gene delivery nor the subsequent gene expression causes any adverse effect on the target cells or tissues. Several experimental tests were conducted to evaluate the lentiviral vector-mediated transduction of cells for potential changes of cellular function including cell morphology, proliferation, and cellular activation in the transcriptional profiling of macrophage-related functional and regulatory genes, and in the releasing of pro-inflammatory cytokines in transduced hMDM.

First, the comparison of transduced and non-transduced cells shows no apparent alternation in cell morphology following the transduction with HR-Hutat2 in both cell lines and primary hMDM (Figure 1A,C). Transduced cell lines were monitored for more than 20 passages, and no change in growth kinetics was observed during that time. In addition, there were no significant differences in cellular viability between normal HTB-11 and HR-Hutat2-transduced HTB-11, as determined by an MTT assay (Figure 3C).

**Figure 6:**
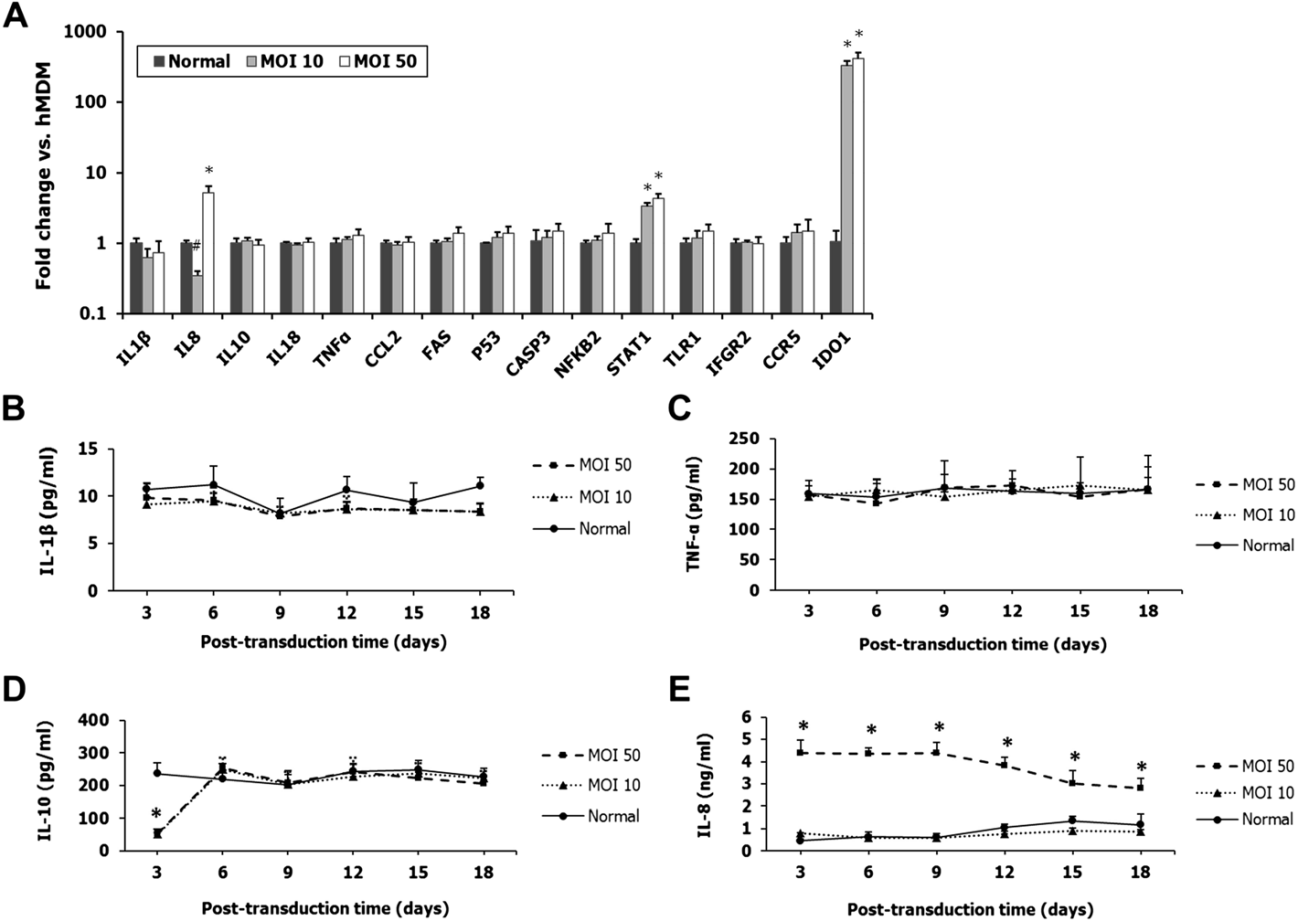
**The effects of transduction with lentiviral vector HR-Hutat2 on the gene expression of human macrophage-related functional and regulatory genes and on kinetics of pro-inflammatory cytokines IL1β, IL8, IL10, and TNF-α.** Human monocyte-derived macrophages (hMDM) were differentiated from isolated peripheral blood mononuclear cells in M-CSF-containing medium. On day 7 and day 8 *in vitro* (DIV 7 and DIV 8), hMDMs were transduced with HR-Hutat2 vector at a MOI of 10 or 50. Total RNA was extracted from non-transduced hMDM (Normal) and transduced hMDM on day 9 post-transduction. Cell culture mediums were collected every 3 days post-transduction. **(A)** Comparative analysis of the transcriptional profiling of 15 hMDM-related functional and regulatory genes by qRT-PCR. Among the 15 genes, only the transcription of *IL8*, *STAT1*, and *IDO1* genes changed. **(B–E)** Sequential changes of IL1β, IL10, IL8, and TNF-α levels in the supernatants of normal and transduced hMDMs at a MOI of 10 or 50. Normal, Non-transduced hMDM; MOI 10, hMDM transduced with HR-Hutat2 at the MOI of 10; MOI 50, hMDM transduced with HR-Hutat2 at the MOI of 50. *P <0.01, ^#^ *P* <0.05 compared with normal. Results shown represent mean values from three independent experiments. Error bars denote the s.e.m.

Second, a qRT-PCR assay was employed to comparatively evaluate the expression of 15 human macrophage-related pro-inflammatory cytokine genes, apoptosis-related genes, tumor-related genes, cell signal transduction genes, and cell surface receptor genes (Table 1) between normal and HR-Hutat2-transduced hMDM on day 9 post-transduction. Differential gene expression was considered “significant” when the normalized fold change of samples versus control was >2 (up-regulated) or <0.5 (down-regulated). Twelve out of 15 genes retained their expression at the same level in transduced hMDM at a MOI of 10 or 50 compared with normal hMDM (Figure 6A). However, the change of gene expression level was detected in three genes, *IL8*, *STAT1*, and indoleamine-pyrrole 2,3-dioxygenase (*IDO)1. STAT1* was 3.36 ± 0.34-fold up-regulated in the MOI 10 group and 4.29 ± 0.77-fold up-regulated in the MOI 50 group as compared to non-transduced hMDM (*P* <0.01). It was 326.8 ± 56.5- and 409.3 ± 86.3-fold up-regulated for *IDO1* gene expression level in transduced hMDM at a MOI of 10 and 50, respectively (*P* <0.01). The expression of *IL8* increased by 5.2 ± 1.2-fold for the transduction at a MOI of 50 (*P* <0.01) as compared to non-transduced hMDM.

Furthermore, to confirm whether the differential gene expression would relate to the protein translation, we sequentially evaluated four pro-inflammatory cytokines, IL1β, IL8, IL10, and TNF-α levels in the conditioned medium of transduced and non-transduced hMDM. Consistently with the results of gene expression profiling, the levels of IL1β and TNF-α in the supernatants of both transduced hMDM groups did not change significantly on each post-transduction day as compared to non-transduced hMDM (Figure 6B,C). The release of IL10 in each transduced hMDM decreased about 4-fold on day 3 post-transduction (51.7 ± 3.6 pg/mL in the MOI 10 group and 54.5 ± 11.2 pg/mL in the MOI 50 group, compared to 236.4 ± 33.5 pg/mL in the non-transduced hMDM group), which returned to normal levels from day 6 post-transduction and maintained these normal levels on each following day (Figure 6D). The IL8 levels in the supernatants were increased on each of the post-transduction days in the MOI 50 group, which was consistent with the up-regulated *IL8* gene expression. However, in the MOI 10 group, although the *IL8* gene expression level was slightly down-regulated, there was no significant change for the secretion of IL8 in the medium compared to the normal control (Figure 6E).

## Discussion

This study had provided evidence for the anti-Tat Hutat2:Fc neutralizing strategy to successfully attenuate HIV-1 Tat-induced neurotoxicity *in vitro*. Specifically, we cloned the *Hutat2:Fc* construct into a lentiviral vector to transduce human cell lines of both neuron and monocyte origins, as well as primary hMDM. Then, we characterized the Hutat2:Fc expression, secretion, and specificity to recognize HIV-1 Tat_86_. The Hutat2:Fc fusion protein not only protected neurons from HIV-1 Tat-induced neurotoxicity, but also protected hMDM against HIV-1_Ba-L_ infection *in vitro.* These results provided proof-of-concept evidence for a novel therapeutic approach against HAND through utilizing hMDM as a therapeutic gene delivery vehicle following transduction with an HIV-1-based lentiviral vector that expresses humanized anti-HIV-1 Tat Hutat2:Fc.

Although the causes of HAND are still unclear, there is a general agreement that the pathogenic mechanisms of HAND are induced by neurotoxic HIV-1 proteins, including Tat, and by pro-inflammatory cytokines and chemokines, as well as NO produced by HIV-1-infected macrophages and glial cells [27-29,34-36,45-47]. Therefore, an ideal therapeutic strategy for HAND is to maximize inhibition of HIV-1 infection and replication in the CNS, as well as to reduce the release of pro-inflammatory products. The current approach for the treatment of HAND is cART [1,48]. However, cART cannot completely prevent HIV replication in the CNS, which is why HIV persists at low-levels in the brain and continues to drive neurodegeneration [1]. Additionally, different antiretroviral agents show variable penetration into the CNS. Although a CPE rank system was formalized in order to improve the efficacy of cART for HAND [7,48], several studies have suggested CPE is a flawed metric. A cART regimen with a high CPE score did not improve neurocognitive performance in individuals with HAND, but rather increased the risk of HIV dementia [10,11]. Thus, adjuvant therapies such as neuroprotective and anti-inflammatory approaches are being explored [1]. To date, adjuvant therapy for HAND is facing three major challenges: which targets to choose, what kinds of reagents to deliver, and how to deliver therapeutic reagents into the CNS.

In this study, the choice of utilizing a humanized anti-HIV-1 Tat scFv Hutat2 as a therapeutic candidate was based on the evidence that Hutat2 had been shown to be highly effective in inhibiting HIV replication both *in vitro* and *in vivo* [22,37-39]. In terms of pathophysiology, both intracellular and extracellular Tat proteins play an important role in the development of HAND. Tat is a potent activator of viral transcription, which promotes the replication of HIV-1 and the production of other neurotoxic viral proteins including gp120 from HIV-1-infected cells [49,50]. Although Tat is normally processed in the nucleus, it is also secreted from infected cells to affect neighboring cells by causing direct neurotoxicity, bystander glial cell activation, and releasing of pro-inflammatory cytokines, chemokines, and NO [28,34-36,51]. HIV-1 Tat proteins from primary virus isolates are encoded by two exons, which consist of 1 to 86 or 1 to 101 amino acids, respectively [52-54]. The LAI/Bru strain of HIV-1 (Clade B) encodes a Tat protein which consists of 1 to 86 amino acids due to a premature stop codon within the second tat exon [55]. HIV-1 Clade C infections are more prevalent in sub-Saharan Africa and Asia, whereas HIV-1 Clade B is the predominant subtype in the USA, Canada, Western Europe, and Australia [56]. In addition, the differences in neurotoxicity of Tat are clade-specific. Several studies have demonstrated that recombinant Tat clade C has an attenuated potential for direct neurotoxicity [57-59] and a decreased ability to induce indirect neurotoxicity [58,60,61]. In a previous study, Tat-transgenic mice were used as an animal model for HAND in which a gene that codes for Tat 1 to 86 amino acids was specifically integrated into astrocytes, producing brain-specific expression [62,63]. In agreement with others and our previous work, Tat_86_ at a concentration of 500 nM or above induced neuron death [24,64]. Thus, in order to evaluate the protective effect of Hutat2:Fc, we used 500 nM of Tat_86_ (Clade B) to produce a dynamic range of cytotoxicity.

An HIV-1-based lentiviral vector is an effective gene transfer system for transducing both dividing and non-dividing cells such as primary cultures of hMDM prepared from human whole blood. To inactivate both the intracellular and extracellular Tat, a self-inactivating HIV-1-based lentiviral vector expressing anti-Tat Hutat2:Fc with a N-terminal IgG leader sequence was used to transduce human cell lines and primary hMDM. In the present study, anti-Tat was produced in the scFv:Fc format as opposed to scFv or to full-length IgG for gene transfer for several reasons. First, the Fc domain folds independently and can improve the solubility and stability of the partner molecule both *in vitro* and *in vivo*, thus remarkably increasing the fusion protein half-life, which prolongs therapeutic activity [65,66]. In addition, the Fc domain can prolong serum half-life by binding to the neonatal Fc receptor [67,68]. Second, the Fc domain can increase the expression and secretion of proteins in mammalian cells to a high level [69,70]. Third, the Fc region allows for easy cost-effective quantification by ELISA which was used in this study and purification by protein-G/A affinity chromatography [66]. Fourth, the small size of the scFv:Fc format may allow greater tissue penetration than a whole IgG [20,71]. The IgG leader in the construct was used to direct the expression of Hutat2:Fc to the endoplasmic reticulum, where Hutat2:Fc can be secreted into cell culture medium more efficiently [22]. As evidenced in this study, the transduction and expression of Hutat2:Fc in HTB-11, U937, and hMDM cells led to detectable high levels of protein in the cell culture medium. In HTB-11 and U937 cells, the *Hutat2:Fc* gene was stably expressed for more than 20 passages and sustained at a high level, reaching to 600 ng/mL in HTB-11 and 33 ng/mL in U937 within a 24-hour cultivation time. Moreover, we confirmed the accumulation of the secreted fusion protein in the culture mediums from these transduced cell lines. Spin-infection was reported as an efficient method to improve the transduction efficiency for cell suspensions [72]. It was noticed that, although the transduction efficiency of monocytic U937 cells was increased to more than 95% after the second-round of spin-infection, the *Hutat2:Fc* gene expression and the protein secretion levels were much lower than those detected from transduced HTB-11 cells. Among transduced HTB-11 and U937 cell lines and primary hMDM, the highest *Hutat2:Fc* transcription level was found in transduced HTB-11 cells, which is 162.5-fold higher than that in transduced hMDM and 18.0-fold higher than that in transduced U937. Similarly, the difference of the concentration of Hutat2:Fc in conditioned medium was also confirmed. This could partly explain why the protection effects of the conditioned medium from transduced hMDM are not as high as those from transduced HTB-11 and anti-Tat antibody *in vitro*. A potential explanation for this difference in protein expression levels is that HTB-11 cells may have a higher integrated copy number of the target gene than myeloid lineage cells, including U937 cell lines and primary hMDM. This is consistent with previous observations that neural cells are more readily transduced by HIV-1-based vectors than cells of myeloid lineage such as macrophages and microglia [24,73]. In addition, the intercellular dNTP level was reported to be vital for HIV-1 reverse transcription and viral replication [74]. However, the concentration of intercellular dNTP in non-dividing macrophages was very low compared to that of dividing cells [75,76]. Thus, the HIV-1-based vector transduction efficiency and the *Hutat2:Fc* gene expression level in primary hMDM were not expected to be as high as those in HTB-11 and U937 cells. Alternatively, it is possible that there may be other intrinsic differences in the ability of different cell types to produce and secrete Hutat2:Fc.

In terms of delivering therapeutic genes into the CNS, there are several candidate methods, including direct invasive injection of viral vectors or genetically modified cells into the cerebrum, which compromise the BBB and produce a reliable gene expression efficiency [77-79]. However, these are not viable therapeutic approaches for HAND in human since they are often accompanied with traumatic brain injuries and repetitive administration may be required. Non-invasive CNS delivery methods are more viable. Circulating monocytes and monocyte-derived macrophages are known to migrate across the BBB and to enter the CNS under normal physiological conditions and certain pathological circumstances [80-84]. Moreover, some of these cells can subsequently mature into long-lived tissue-resident brain macrophages and microglia [84,85]. Thus, monocytes/MDMs have the potential to deliver therapeutic reagents or genes into the CNS as “Trojan horses” [86]. Some advantageous attempts have been made for the treatment of neurodegenerative diseases including HAND. For example, it was reported that genetically-modified circulating CD11b^+^ cells (largely monocytes) were used to deliver and express the protease neprilysin gene into the CNS to arrest amyloid deposition in an Alzheimer’s disease transgenic murine model [82]. Genetically-modified macrophages were utilized to deliver glial cell-derived neurotrophic factor for the treatment of Parkinson’s disease in a murine model [87]. Nanoformulated antiretroviral drugs were also delivered into the brain by MDMs in a murine model of HAND [80]. Thus, in this study, we explored a promising therapeutic strategy through the use of MDMs as a potential gene delivery vehicle.

We demonstrated that lentiviral vector-mediated gene transfer could be successfully used in hard-to-transduce monocytic cell lines such as U937 and primary hMDM, which led to stable expression of Hutat2:Fc fusion protein. Not only was the expression stable at a high level over time, but also the secreted Hutat2:Fc from different transduced cells was shown to be consistently biologically active. DIBA analysis and Western blotting demonstrated that the secreted Hutat2:Fc bound directly to HIV-1 Tat_86_ as a full-length anti-Tat monoclonal antibody, whereas the A3H5:Fc control could not. In addition, Hutat2:Fc expressed from lentiviral vector-transduced HTB-11 or hMDM (at final concentrations of 536 ng/mL for HTB-Hutat2 and 42.8 ng/mL for hMDM-Hutat2) conferred significant neuroprotection against neurotoxicity induced by HIV-1 Tat_86_ in the human neuronal cell line HTB-11 and primary murine neuron culture. Moreover, it has been reported that although anti-Tat antibody could not fully block HIV infection, it could suppress HIV replication [88-90]. As shown in this study, Hutat2:Fc in conditioned medium from hMDM-Hutat2 at a final concentration about 106.9 ng/mL was able to suppress HIV-1_Ba-L_ replication in primary hMDM. Additionally, HR-Hutat2-transduced hMDM presented resistance against viral replication. These findings suggest that delivery of genetically-modified primary MDM expressing Hutat2:Fc to the CNS to attenuate neuro-inflammation, suppress HIV-1 replication, and reduce the spread of viral infection would be a very promising therapeutic strategy against HIV-1 Tat-induced neurotoxicity. However, it should be noticed that the production of Hutat2:Fc in transduced hMDM was not as high as in transduced neuronal HTB-11 cells. The production of lower amounts of Hutat2:Fc protein reduced the neuroprotective effect. Furthermore, it is unclear how efficiently transduced MDM would get into the CNS and how many transduced MDM would be necessary to produce a significant effect on the development of neuropathology. Another limitation of this study is that the HIV challenge experiment was an acute HIV infection *ex vivo*. We did not evaluate the effect of Hutat2:Fc on viral suppression in a chronic HIV infection model, especially when the virus was already suppressed by antiretroviral regimens. Further animal studies will be needed to explore these issues.

The self-inactivating lentiviral vector-based gene therapy is relatively safe and some vectors are currently being evaluated in clinical trials [91]. Our findings also showed that the transduced cell line HTB-11 did not result in any measurable alternation in cell viability. However, MDM, considered as plastic cells, are double-edged swords for anti-infectious immunity as well as tissue injury and repair. As with T cells, monocytes can be activated and polarized into either the classically activated pro-inflammatory (M1) macrophages subtype, or an anti-inflammatory alternatively activated (M2) subtype according to their micro-environments [92-94]. Defining macrophages based on their specific functional activities is a more appropriate approach [94]. Granulocyte macrophage colony stimulating factor (GM-CSF) and M-CSF are involved in the differentiation of monocytes to macrophages [92,93]. Specifically, GM-CSF causes initial differentiation of monocytes towards the M1 macrophage subtype with a pro-inflammatory cytokine profile (e.g., TNF-α, IL1β, IL6, IL23), whereas M-CSF treatment produces an anti-inflammatory cytokine (e.g., IL10, TGF-β) profile similar to M2 macrophages [92,93]. Our findings also confirmed that M-CSF stimulated the monocytes within the peripheral blood mononuclear cell population differentiation toward an M2-like phenotype with a high production of IL10 (Figure 6C), which would be more beneficial to the CNS wound healing. However, this polarization can be switched to an M1-like phenotype under the circumstance of acute microbe infection [95]. Thus, we investigated the potential immune-activation induced by lentiviral vector transduction. Our results indicated that the gene expression level of eight immune-related genes, including *IL1β*, *IL10*, *IL18*, *TNF-α*, *CCL2*, *TLR1*, *IFGR2*, and *CCR5*, and four cell cycle regulator, apoptosis, and signal transduction-related genes, including *Fas*, *P53*, *CASP3*, and *NFKB2*, in 15 candidate genes were not significantly changed following transduction as compared to non-transduced hMDM (Figure 6A). No change was observed in the concentrations of IL1β and TNF-α after transduction, which further confirmed the results of qRT-PCR (Figure 6B,C). Transduction with HR-Hutat2 resulted in a dramatical decrease of IL10 production on day 3 post-transduction (Figure 6D). However, this change recovered from day 6 post-transduction and other cytokines related to M1 polarization state, such as IL1β and TNF-α, did not significantly change during the following days (Figure 6B–D). This means that lentiviral transduction induced a transient decrease of IL10 production, but did not completely switch the polarization of hMDM from the M2 to M1 phenotype.

However, we also found some atypical M1-skewed polarization profiles in response to lentiviral transduction. Notably, three genes, including *IL8*, *STAT1*, and *IDO1*, were up-regulated in transduced hMDM at a MOI of 50 (Figure 6A). Although the *IL8* mRNA expression was down-regulated, the release of IL8 did not change in transduced hMDM at a MOI of 10 (Figure 6E). IL8 is a pro-inflammatory cytokine, which induces phagocytosis and chemotaxis in target cells, primarily neutrophils, and also other granulocytes, causing them to migrate toward the site of infection. STAT1 is a member of the signal transducers and activators of transcription family, which up-regulated when macrophage polarized toward an M1 phenotype [96]. IDO encoded by *IDO1* gene is the rate-limiting enzyme of tryptophan catabolism through the kynurenine pathway, thus causing depletion of tryptophan. It has been reported that *IDO1* gene expression was up-regulated and IDO activity was increased in HIV-1 simian immunodeficiency virus (SIV)-, and feline immunodeficiency virus-infected T cells as well as macrophages [97-100]. Moreover, HIV-1 Tat was proved to increase expression of IDO in murine organotypic hippocampal slice cultures and in human primary astrocytes [101,102]. IDO activation was related to the modulation of the immune response and neuropathogenic effects in HIV infection. For example, several findings suggested that an increase of functional IDO enzymatic activity is correlated with immunosuppression by its ability to inhibit lymphocyte proliferation and with increased production of neurotoxins, such as kynurenine and quinolinic acid, in the brain [97,103-105]. In SIV-infected macaques, mRNA expression of cytotoxic T lymphocytes antigen-4 (CTLA-4) and FoxP3, markers of regulatory T cells (T_reg_), as well as IDO, were increased in the spleens, mesenteric lymph nodes, colons, and jejuna, and were directly correlated to SIV RNA in the same tissues [99]. CTLA-4 blockade decreased IDO and viral RNA expression, and increased the effector function of both SIV-specific CD4^+^ and CD8^+^ T cells in lymph nodes [106]. Inhibition of IDO activity led to enhanced generation of HIV-1-specific cytotoxic T lymphocytes, leading to elimination of HIV-1-infected macrophages in the CNS [103]. These data indicated increased IDO expression or activity might favor HIV/SIV replication and the establishment of viral reservoirs in lymphoid tissues and in the CNS. However, a few studies showed inconsistent effects regarding the up-regulated IDO expression on viral replication. Although IDO transcripts were increased in HIV encephalitis, IDO activation would likely suppress intracellular viral replication in astrocytes [107]. IDO function probably dissociated from protein expression, which would be determined by the local CNS cytokine and NO microenvironment [107]. A recent study found that the up-regulation of *IDO1* mRNA expression was likely contributed to macrophage M1 polarization [93]. Furthermore, M1 polarization of hMDM would restrict HIV-1 replication in pre- and post-integration steps [108]. Hence, the role of IDO in HIV-induced inflammation of the CNS was not completely clear and probably double-edged. In this study, the HIV-1-based lentiviral vector also induced an up-regulated *IDO1* gene expression in hMDM. Moreover, similar gene expression profiling was found in both HR-Hutat2-transduced hMDM at the different MOIs and HR-A3H5-transduced hMDM (data not shown). These findings indicated that the up-regulation of *IDO1* gene expression was induced by a vector transduction process independently, and not due to the presence of Hutat2:Fc. Although vector transduction promoted the expression of *IDO1* gene and stimulated hMDM polarization towards atypical M1-skewed polarization profiles, the functions of IDO and M1-skewed profiles in neuropathogenesis and viral remission were microenvironment-dependent and require further investigation. In addition, our current study did not observe any significant neurotoxicity from the conditioned mediums in the neuron protection assay. In other words, the neuron protective effects of Hutat2:Fc probably have overpowered the potential side effects induced by lentiviral vector transduction.

To conclude, this study provides a preliminarily functional evaluation of anti-HIV-1 Tat Hutat2:Fc and transduced cells against Tat_86_-induced neurotoxicity and HIV-1 challenge *in vitro*. Further investigations on *in vivo* neuronal protection and HIV-1 inhibition of transduced monocytes/macrophages for gene delivery into the CNS are required. On the other hand, the vector transduction induced alternation on the expression of several genes, including *IL8*, *STAT1*, and *IDO1*, presenting potential immunological effects on transduced macrophages and the clearance of virus in the CNS. Thus, examining the potential side effects of exploring this technology as a therapeutic strategy in HAND animal models is definitely essential for future studies.

## Conclusions

Our study demonstrated that an HIV-1-based lentiviral vector could efficiently transfer therapeutic the anti-HIV-1 Tat *Hutat2:Fc* gene into human neuronal and monocytic cell lines as well as primary cultures of hMDM. Hutat2:Fc can be stably expressed and secreted from the transgenic cells and can protect neurons against HIV-1 Tat_86_-induced neurotoxicity, and suppress but not fully block HIV-1_Ba-L_ replication in both non-transduced and transduced hMDM *in vitro*. Moreover, lentiviral transduction did not result in any significant changes in cytomorphology and cell viability. Although the expression of *IL8*, *STAT1*, and *IDO1* genes was up-regulated in transduced hMDM, such alternation in these gene expression profiles did not affect the neuroprotective effect of Hutat2:Fc. While much work is still required to develop a viable approach for application in patients, these findings provide interesting insights for utilizing *Hutat2:Fc* gene-modified monocytes/macrophages as a potential novel therapeutic strategy for HAND.

## Additional files

Additional file 1:
**Schematic map of the HIV-1-based transfer plasmid.** The HIV-1-based lentiviral vector was used to express enhanced green fluorescent protein (EGFP), with either the therapeutic anti-HIV-1 Tat single chain fragment intrabody (scFv) Hutat2:Fc fusion protein (HR-Hutat2), or the control scFv A3H5:Fc fusion protein (HR-A3H5); the fusion proteins used the human IgG leader to direct the expression to the endoplasmic reticulum and used the Fc domain to increase stability and to tag protein expression. LTR, Long terminal repeat; ψ, Packaging signal; SD, Splice donor; SA, Splice acceptor; CMV, Cytomegalovirus promoter; scFv:Fc, The construct encoding the anti-Tat Hutat2 fused to Fc or the anti-Epstein-Barr virus latent membrane protein 1 (LMP-1) A3H5 fused to Fc; Fc, Hinge domain from IgG1 and the Fc domain from human IgG3; IRES, Internal ribosome entry site; GFP, Green fluorescent protein. Primers used for molecular cloning: forward/reverse, 5′-CCG*CTCGAG*CGGGCCGGCCATGGCCCAGGTGCA-3′/5′-CGC*GGATCC*GCGTTAAATCATTTACCCGGAGACAGG-3′ (italics indicate the restriction enzyme cutting site). Additional file 2:
**CD14 staining for primary culture of hMDM.** After three washings with PBS, primary culture of hMDM was stained with a human CD14 monoclonal antibody conjugated with R-phycoerythrin on day 6 *in vitro* (DIV 6). The purity of hMDM culture *in vitro* was calculated to be >98%. Additional file 3:
**Specific binding of Hutat2:Fc from transduced cells to HIV-1 Tat**_**86**_ **by Western blot assay.** HIV-1 Tat_86_ (14 kDa) was separated by SDS-PAGE electrophoresis and transferred onto NCM. Each NCM was incubated with the conditioned mediums from HR-Hutat2-transduced cells (HTB-Hutat2, U937-Hutat2, and hMDM-Hutat2) at 4°C overnight followed by incubation with rabbit anti-human IgG_(H+L)_ and goat anti-rabbit IgG-HRP conjugated antibodies, respectively. Specific binding was visualized by the color deposition on the NCM when DAB was added. The Tat-containing NCM incubated with the conditioned medium from HR-A3H5-transduced HTB-11 served as a negative control (HTB-A3H5), while the Tat-containing membrane incubated with rabbit anti-Tat serum served as a positive control (Pos Ctl). The lane loaded with Tat dilution buffer was used as a blank control (BLK Ctl).

## Abbreviations

A3H5:Fc: Anti-Epstein-Barr virus latent membrane protein 1 scFv A3H5:Fc fusion protein
BBB: Blood-brain barrier
BSA: Bovine serum albumin
cART: Combined antiretroviral therapy
CNS: Central nervous system
CPE: CNS penetration-effectiveness
CTLA-4: Cytotoxic T lymphocytes antigen-4
DAB: 3,3-diaminobenzidine tetrahydrochloride
DIBA: Dot-immunobinding assay
DIV: Days *in vitro*
ELISA: Enzyme-linked immunosorbent assay
FBS: Fetal bovine serum
GM-CSF: Granulocyte macrophage colony stimulating factor
HAND: HIV-associated neurocognitive disorder
hMDM: Human monocyte-derived macrophages
HRP: Horseradish peroxidase
Hutat2:Fc: Humanized anti-Tat scFv:Fc fusion protein
IDO: Indoleamine-pyrrole 2,3-dioxygenase
IRES: Internal ribosome entry site
MAP2: Microtubule-associated protein 2
M-CSF: Macrophage colony stimulating factor
MDM: Monocyte-derived macrophages
MOI: Multiplicity of infection
NCM: Nitrocellulose membrane
NO: Nitric oxide
RT: Room tempreature
scFv: Single-chain variable fragment intrabodies
SDS: Sodium dodecyl sulfate
TBST: Tris-buffered saline containing 0.05% Tween 20
T_reg_: Regulatory T cells
TUNEL: Terminal dexoynucleotidyl transferase-mediated dUTP nick end labeling
