# Supplementary figures and images for: Anti-tat Hutat2:Fc mediated protection against tat-induced neurotoxicity and HIV-1 replication in human monocyte-derived macrophages
Source: J Neuroinflammation. 2014 Nov 22;11:195. doi: 10.1186/s12974-014-0195-2 (PMC4256057; doi:10.1186/s12974-014-0195-2)

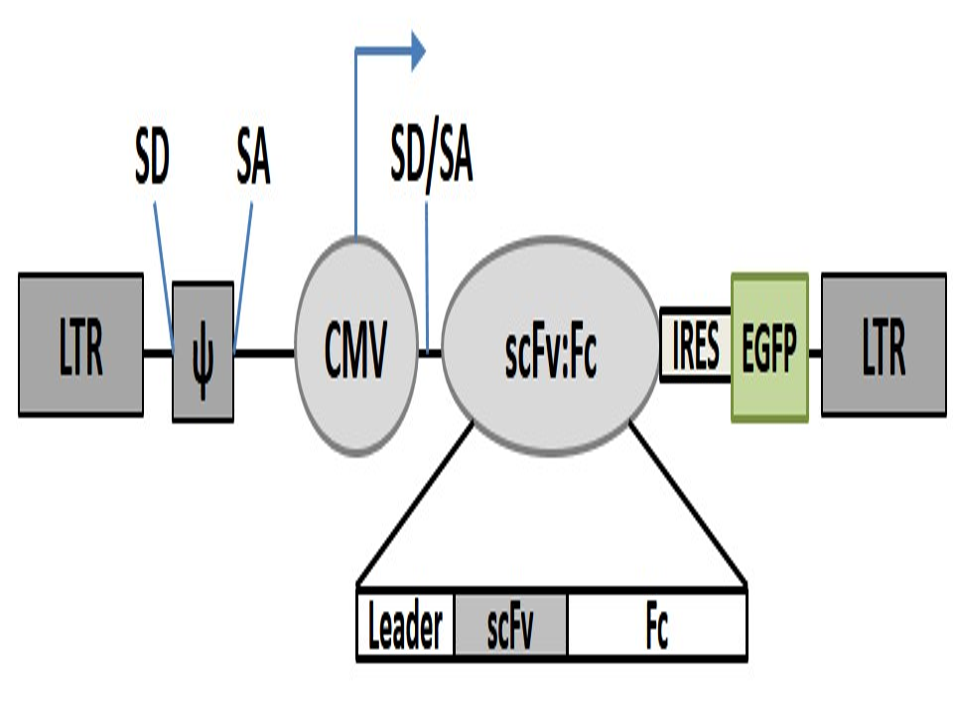

Supplement: Additional file 1: — Schematic map of the HIV-1-based transfer plasmid. The HIV-1-based lentiviral vector was used to express enhanced green fluorescent protein (EGFP), with either the therapeutic anti-HIV-1 Tat single chain fragment intrabody (scFv) Hutat2:Fc fusion protein (HR-Hutat2), or the control scFv A3H5:Fc fusion protein (HR-A3H5); the fusion proteins used the human IgG leader to direct the expression to the endoplasmic reticulum and used the Fc domain to increase stability and to tag protein expression. LTR, Long terminal repeat; ψ, Packaging signal; SD, Splice donor; SA, Splice acceptor; CMV, Cytomegalovirus promoter; scFv:Fc, The construct encoding the anti-Tat Hutat2 fused to Fc or the anti-Epstein-Barr virus latent membrane protein 1 (LMP-1) A3H5 fused to Fc; Fc, Hinge domain from IgG1 and the Fc domain from human IgG3; IRES, Internal ribosome entry site; GFP, Green fluorescent protein. Primers used for molecular cloning: forward/reverse, 5′-CCGCTCGAGCGGGCCGGCCATGGCCCAGGTGCA-3′/5′-CGCGGATCCGCGTTAAATCATTTACCCGGAGACAGG-3′ (italics indicate the restriction enzyme cutting site). [file 12974_2014_195_MOESM1_ESM.tiff]

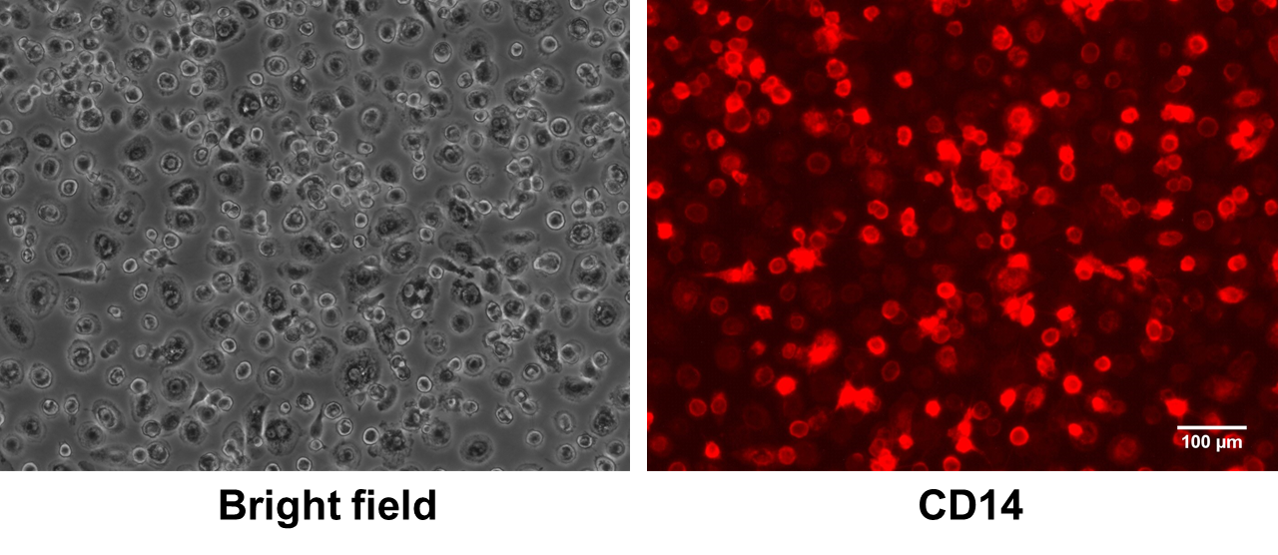

Supplement: Additional file 2: — CD14 staining for primary culture of hMDM. After three washings with PBS, primary culture of hMDM was stained with a human CD14 monoclonal antibody conjugated with R-phycoerythrin on day 6 in vitro (DIV 6). The purity of hMDM culture in vitro was calculated to be >98%. [file 12974_2014_195_MOESM2_ESM.tiff]

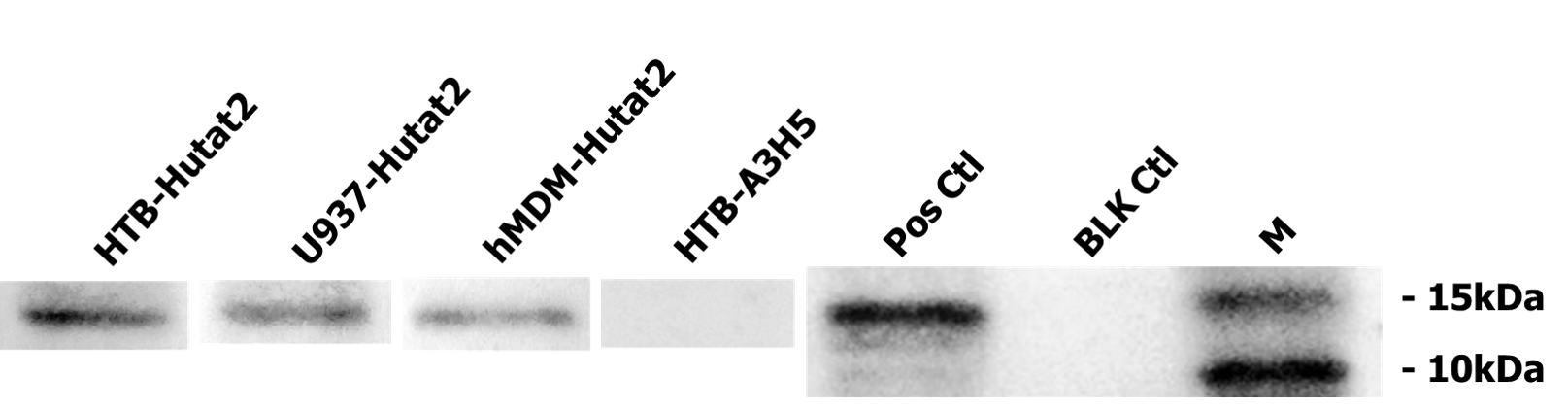

Supplement: Additional file 3: — Specific binding of Hutat2:Fc from transduced cells to HIV-1 Tat 86 by Western blot assay. HIV-1 Tat86 (14 kDa) was separated by SDS-PAGE electrophoresis and transferred onto NCM. Each NCM was incubated with the conditioned mediums from HR-Hutat2-transduced cells (HTB-Hutat2, U937-Hutat2, and hMDM-Hutat2) at 4°C overnight followed by incubation with rabbit anti-human IgG(H+L) and goat anti-rabbit IgG-HRP conjugated antibodies, respectively. Specific binding was visualized by the color deposition on the NCM when DAB was added. The Tat-containing NCM incubated with the conditioned medium from HR-A3H5-transduced HTB-11 served as a negative control (HTB-A3H5), while the Tat-containing membrane incubated with rabbit anti-Tat serum served as a positive control (Pos Ctl). The lane loaded with Tat dilution buffer was used as a blank control (BLK Ctl). [file 12974_2014_195_MOESM3_ESM.tiff]
